# Impact of China’s National Volume-Based Procurement on Drug Procurement Price, Volume, and Expenditure: An Interrupted Time Series Analysis in Tianjin

**DOI:** 10.34172/ijhpm.2023.7724

**Published:** 2023-09-20

**Authors:** Boya Zhao, Jing Wu

**Affiliations:** ^1^School of Pharmaceutical Science and Technology, Tianjin University, Tianjin, China; ^2^Center for Social Science Survey and Data, Tianjin University, Tianjin, China

**Keywords:** Drug procurement, Centralized, Volume-based, Price, Volume, China

## Abstract

**Background:** National Volume-Based Procurement (NVBP) program has been carried out in China to lower drug prices and reduce patients’ medication burden. This study aims to evaluate its impact on drug purchasing in Tianjin city, one of the first 11 cities piloting NVBP in China.

**Methods:** Using monthly drug procurement data from Tianjin Medical Purchasing Center between 2018 and 2020, this study identified bid-winning drugs and their alternative drugs in the pilot NVBP, and evaluated the policy impacts on their procurement price (cost of defined daily dose, DDDc), volume (the number of defined daily dose, DDDs), and expenditure, during the first (initiated at April 1, 2019) and second (initiated at April 25, 2020) procurement cycles of pilot NVBP, applying interrupted time series (ITS) analysis. Included drugs were classified into 12 pharmacological subgroups for further analysis.

**Results:** Decrease in DDDc of NVBP-covered drugs (bid-winning and non-winning drugs) were observed in the first (level change: -CNY 3.878/DDD, *P*<.001; trend change: -CNY 0.068/DDD, *P*=.001; relative change: -61.55%) and second (level change: -CNY 0.356/DDD, *P*=.049) procurement cycles of pilot NVBP, while no significant change was observed for the DDDc of alternative drugs, except for the increase in antidiarrheic and anti-inflammatory/antirheumatic subgroups as more expensive drugs were purchased from new suppliers in the second procurement cycle. The DDDs of bid-winning drugs significantly increased, while decreased for the non-winning original and generic drugs. Procurement expenditure was saved for NVBP-covered drugs (level change: -CNY 7.29×107, *P*<.001; trend change: -CNY 5.62×106, *P*<.001; relative change: -62.60%). However, during the second procurement cycle, procurement volume and expenditure of alternative drugs increased significantly in 7 out of 12 subgroups.

**Conclusion:** The pilot NVBP policy in China reduced procurement price, promoted generic substitution, and saved procurement expenditure. However, the increase in procurement price, volume and expenditure of alternative drugs may reveal the significance of regulating healthcare institutions’ drug purchasing behavior.

## Background

**Key Messages**

**Implications for policy makers**

- By creating economies of scale and improving efficiency, the National Volume-Based Procurement (NVBP) in China effectively reduced procurement price and saved procurement expenditure.
- However, the increases in procurement price, volume, and expenditure of alternative drugs that are not covered by the policy but are clinically substitutable for the bid-winning drugs indicated that healthcare institutions might have a tendency to seek extra finical incentives, which might diminish the overall benefits of NVBP.
- The experience of China’s NVBP underscores the importance of deliberate systematic thinking in the design and implementation of centralized drug procurement (CDP), which calls for taking into the broader implications of the policy beyond its primary targets.
- It is crucial to consider the diversity and substitutable relationship among drugs within the same pharmacological or therapeutic areas, when designing a CDP policy.
- Establishing a long-term, sustainable, and systematic system to regulate the procurement of the bid-winning and alternative drugs, complemented by efficient financial incentive steering measures, seem necessary.

**Implications for the public**

Through lowering drug procurement prices and promoting generic substitution, the National Volume-Based Procurement (NVBP) policy has improved the affordability and accessibility of drugs across various pharmacological areas for patients, and these policy effects have demonstrated sustainability over time. However, the increase in procurement price, volume, and expenditure of alternative drugs may diminish the policy benefits, leading to unnecessary frequent transitions between the bid-winning drugs and alternative drugs for patients or increases in economic burden. Moreover, as the policy progressed, this problem became increasingly prevalent, potentially affecting more patients. Appropriate approaches should be designed to guide and regulate healthcare institutions’ procurement behavior, ensuring the provision of needed and cost-effective drugs to patients.

Centralized drug procurement (CDP) cooperatively combines the resources of drug purchasing authorities to improve efficiency and create greater purchasing power, which has been widely used by many countries and international organizations as a pharmaceutical pricing policy to achieve affordability and accessibility of pharmaceutical products to patients and healthcare systems.^1,2^

 China applied the national-level CDP since the end of 2018, which is also known as “National Volume-based Procurement (NVBP)” and promoted by the National Healthcare Security Administration (NHSA).^3^ The Joint Procurement Office was established to organize the tendering process and acts on behalf of all local drug procurement agencies and healthcare institutions.^4^ For each drug covered by NVBP (defined by International Nonproprietary Name [INN], administration route and specification), the agreed-upon procurement volume is provided, which is estimated as a percentage of the previous annual usage volumes of healthcare institutions. Compared to previous provincial-level drug procurement without agreed-upon procurement volume, NVBP is more conducive to creating economies of scale. To be eligible for NVBP bidding, generic drugs must pass the bioequivalence testing to verify they are equivalent to their brand-name counterparts, which was previously not strictly required. In the procurement cycle, healthcare institutions are obligated to purchase the bid-winning drugs in quantities that at least meet the agreed-upon procurement volumes, while healthcare institutions also have the option to purchase non-winning drugs with the same INN from other manufacturers. Therefore, it is anticipated that NVBP will not only lead to a reduction in drug prices and save procurement expenditures, but will also improve quality of generic drugs and guide healthcare institutions’ drug purchasing and usage behavior, et al.^3,5^ The first pilot NVBP was initiated in 11 cities across China in 2019, covering 25 drugs. The predetermined percentages for estimating the agreed-upon procurement volume ranged between 60%-70%. In the pilot NVBP, the manufacturer that offered the lowest bidding price of each drug was granted exclusive sale authority within the one-year procurement cycle, and 23 out of 25 bid-winning drugs were generic.

 Many previous studies have shown that CDP can effectively lower the prices and procurement expenditures of bid-winning drugs, including CDP conducted by international organizations such as Eastern Caribbean Drug Service,^6^ Global Fund to Fight AIDS, Tuberculosis and Malaria,^7,8^ or countries such as France,^9^ India,^10^ Brazil,^11,12^ Colombia,^13^ as well as NVBP in China.^4^ Some of these studies suggested that the impacts may vary across time or pharmacological areas, but reliable evidence is scarce, since most of the research periods were shorter than one complete procurement cycle, or did not distinguish or only focused on one specific pharmacological area. In addition, China has highly fragmented drug manufacturing sectors,^14^ for many bid-winning drugs, there are non-winning drugs with the same INN produced by many other manufactures. Moreover, the NVBP only covers limited numbers of drugs, there are lots of alternative drugs not covered by NVBP in the same pharmacological areas. The transaction process of these drugs is less transparent than the bid-winning drugs, which may provide financial incentives for healthcare institutions to purchasing them. Previous studies have observed decrease in procurement volume of non-winning drugs after NVBP, along with the increase for bid-winning drugs.^15-17^ However, the policy effect on the alternative drugs not covered by NVBP is still unclear, with limited studies reporting inconsistent results for drugs in different pharmacological areas such as antibiotic, antihypertensive and antiviral drugs.^16-18^

 Tianjin is one of the first 11 cities piloting NVBP in China, and also one of the four municipalities (provincial cities) in China. After the Joint Procurement Office announced the bid-winning manufactures and prices for 25 drugs covered by the pilot NVBP, Tianjin officially implemented the pilot NVBP at April 1, 2019 and started the first 1-year procurement cycle (Figure S1, Supplementary file 1), with all public healthcare institutions participated. The second procurement cycle of the pilot NVBP in Tianjin started at April 25, 2020, following the guidance of local medical insurance bureau. During the second procurement cycle, the bid-winning prices further dropped nearly for all drugs, and three out of 25 drugs’ bid-winning manufactures changed (Table S1). The second procurement cycle was set to 1 or 2 years for different bid-winning drugs.

 This study aimed to evaluate the impact of the pilot NVBP on the procurement price, volume, and expenditure for the drugs covered by the policy, and the drugs not covered but may affected by the policy, taking Tianjin as an example. Differences among procurement cycles and pharmacological categories were also investigated.

## Methods

###  Setting

 The pilot NVBP policy covered 11 cities including 4 municipalities and 7 sub-provincial cities. In the current study, the impact of the pilot NVBP was investigated in the municipality of Tianjin, which is located in Northern China and covers an area of 11 966 km^2^ with 16 districts.^19^ In 2019, Tianjin accommodated 15.62 million population with a per-capita gross domestic product of CNY 90 371, 127.48% of the national average gross domestic product level.^19,20^ There were 5962 healthcare institutions in Tianjin in 2019, among which 441 were hospitals and 5 348 were healthcare institutions at the basic level.^19^

###  Data Source and Sample Selection

 Data were obtained from Tianjin Medical Purchasing Center upon on the approval from Tianjin Municipal Medical Insurance Bureau for research purposes. The extracted data contain monthly aggregated drug purchase records of healthcare institutions in Tianjin, from January 2018 through December 2020, which included drug’s generic name, brand name, form, specification, package size, manufacture, drug approval number, procurement unit, price per unit, procurement volume, procurement expenditure, procurement date (month-year), and name of the buyer (healthcare institution). The extracted data comprised approximately 970 thousand records, covering 383 INN of the NVBP-covered drugs and alternative drugs (as defined in the subsequent text) in the NVBP policies implemented in Tianjin from 2018 to 2020 (Figure S1).

**Figure 1:**
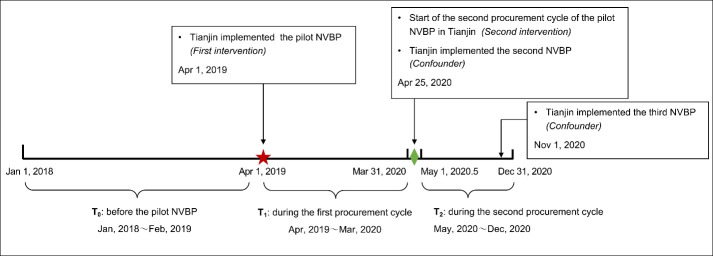
Classification of Study Samples. Abbreviation: NVBP, National Volume-Based Procurement.

 Two interventions, the pilot NVBP initiated on April 1, 2019, in Tianjin, and the start of its second procurement cycle on April 25, 2020, divided the 36 months study period into 3 segments (Figure 1): before the pilot NVBP (T_0_: January 2018 to February 2019), during the first procurement cycle (T_1_: April 2019 to March 2020), and during the second procurement cycle (T_2_: May 2020 to December 2020). Data for March 2019 was excluded because some healthcare institutions purchased the bid-winning drugs at the bid-winning prices in this month before the formal launch of the pilot NVBP. Data for April 2020 were also excluded since the records in the first and second procurement cycles cannot be separated in the monthly aggregated data.

**Figure 2:**
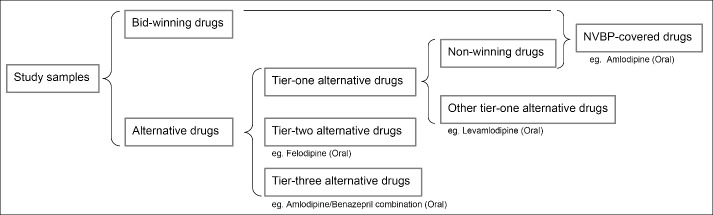
Classification of Study Samples. Abbreviation: NVBP, National Volume-Based Procurement.

 The study samples were the bid-winning drugs in the pilot NVBP and their corresponding alternative drugs. The information of the bid-winning drugs was listed in Table S1. The drugs with the same INN and administration route as the bid-winning drugs were referred as “NVBP-covered drugs,” which included both the bid-winning drugs and non-winning drugs. Alternative drugs refer to the drugs that are clinically substitutable for bid-winning drugs, which were certified by NHSA in the officially issued document “Monitoring Plan Work of National Centralized Drug Procurement and Use” (Table S2). As shown in Figure 2, alternative drugs were classified into three layers, wherein the tier-one alternative drugs were those have the same INNs or similar molecular structures as the bid-winning drugs, the tier-two alternative drugs mainly were those in the same chemical subgroups as the bid-winning drugs, and the tier-three alternative drugs mainly were those treating the same diseases as the bid-winning drugs. However, not all drugs meeting the above criteria were included and classified, taking into account the regulatory consideration and clinical perspective. Therefore, the tier-one alternative drugs exhibit the highest substitutability for the bid-winning drugs, followed by the tier-two and tier-three alternative drugs.

 During the study period, study samples that may have been affected by confounding policies were additionally excluded from this study such as alternative drugs (except the non-winning drugs) with the same INN as the bid-winning drugs in the pilot or second or third NVBP, to ensure that the measured effects of NVBP on alternative drugs were not confounded by its effects on the bid-winning drugs, as well as the antineoplastic alternative drugs that were newly added to the National Reimbursement Drug List through pricing negotiation.

###  Measurement

 Study outcomes of interest were the changes in procurement price, volume, and expenditure of samples. Procurement expenditure was reported in monetary value. Procurement volume was measured as the number of defined daily doses (DDDs) in order to compare drugs with different forms. DDD refers to the average maintenance dose per day for a drug used for its main indication in adults. DDD was uniquely assigned per route of administration within an anatomical therapeutic chemical (ATC) code when the information is directly available on the World Health Organization (WHO) website.^21^ Otherwise, DDD was calculated based on drugs’ instructions following the WHO’s principle for DDD assignment.^21^ The cost of defined daily dose (DDDc), a proxy indicator for procurement price, was computed as “procurement expenditure/DDDs.” The DDDc of both the bid-winning and non-winning drugs were not reported in this study as some of them were not purchased in some months, resulting in the inability to calculate their DDDc values.

###  Statistical Analysis

 Monthly procurement price (DDDc), volume (DDDs), and expenditure were calculated in the study period. The mean values were calculated and compared during the common months (May to December) before and after the two interventions. Single-group interrupted time series (ITS) analyses with two interventions were conducted to evaluate the impact of the pilot NVBP on DDDc, DDDs, and expenditure, in the first and second procurement cycles. Segment regression models were used to measure the level change (change at the first month following the intervention) and trend change (monthly change) after the interventions with level and trend before the interventions controlled:


$$Y_{t}=\beta_{0}+\beta_{1}.\;Time_{t}+\beta_{2}.\;I_{tj}+\beta_{3}.\;Time_{t}.\;I_{tj}+\beta_{4}.\;I_{tk}+\beta_{5}.\;Time_{t}.\;I_{tk}+\sum_{i=1}^{3}\beta_{5+i}.\;D_{i}+\varepsilon_{t}$$

Where *Y*_t_ indicates the outcome of interest measured at time *t*. *Time*_t_ is a continuous variable indicating the months from the start of study period at time *t*. *I*_tj_ and *I*_tk_ are dummy variables indicating the relationship between time *t* and the interventions (the start of the first and second procurement cycles of the pilot NVBP). *I*_tj_ and *I*_tk_ are set to 1 after the interventions, respectively, and 0 in the pre-intervention period. *D*_i_ are three dummy variables that refer to the month of the Spring Festival in 2018, 2019, and 2020 to control the influence of Spring Festival on drug consuming behavior. *ε*_t_ is an estimate of the random error. Therefore, coefficient *β*_2_ represents the level change and *β*_3_ represents the trend change of the chosen outcomes during the first procurement cycles of the pilot NVBP, while the effects in the second procurement cycle are indicated by *β*_4_ and *β*_5_ similarly. Autocorrelation was assessed using plots of autocorrelation and partial-autocorrelation function as well as Durbin-Watson test. The autocorrelation form of the autoregressive–moving-average ARMA (p, q) model for the stationary series were determined. Then the significant autoregressive parameters (p, q) were included in regression models for autocorrelation adjustment.^22-24^ The definitions of the bid-winning drugs in the regression models were consistent with those in Table S1.

 The relative changes in outcomes at the sixth month after the interventions were calculated by comparing predicted outcomes in the presence and absence of the interventions, which combined the level and trend changes.^22,25^

 Subgroup analyses were performed to explore whether the overall impact was consistent in drugs in different pharmacological groups. Twelve pharmacological subgroups were created according to ATC-3 codes and indications of winning products: namely antihypertensives, lipid-modifying drugs, psycholeptics, psychoanaleptics, antiepileptics, antineoplastics, antivirals, antibacterials, antithrombotics, drugs for obstructive airway diseases, antidiarrheics, and anti-inflammatory/antirheumatic drugs (Table S3).

 STATA 15.0 was used for data preparation and R 3.6.1 was used for regression analysis. The significance level was set as two-sided α < 0.05.

## Results

###  Changes in Procurement Price ( DDDc )

 A total of 25 NVBP-covered drugs, and 73 alternative drugs with different INN from the NVBP-covered drugs were included in the analysis (Tables S1 and S2). Descriptive analyses showed the monthly average DDDc of NVBP-covered drugs from May to December 2018, 2019, and 2020 were CNY6.42, CNY2.58, and CNY1.88 per DDD, respectively, demonstrating a decreasing trend. While the corresponding descriptive DDDc for other tier-one, tier-two, and tier-three alternative drugs all revealed the contrary trends (Table S4).

**Figure 3:**
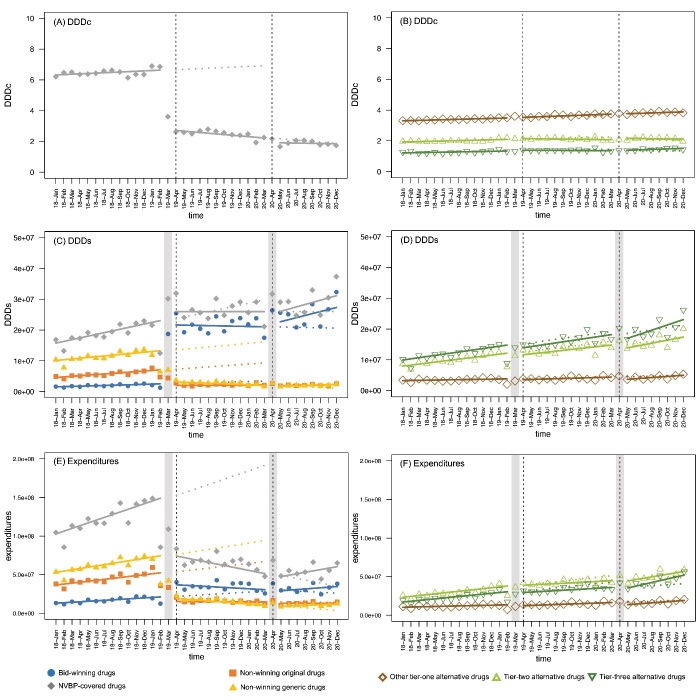
Procurement Price, Volume, and Expenditure Before and After the Pilot NVBP. Abbreviations: NVBP, National Volume-Based Procurement, DDDs, defined daily doses; DDDc, cost of defined daily dose. Note: 1. Shaded areas represented that data in these month were excluded in this study. Vertical dashed lines represented the initiation of the first/second procurement cycle. 2. Solid lines were fitted based on actual values. Dotted lines simulated the counterfactual scenario where the intervention was not implemented.

 The results from the ITS analyses showed that, before the pilot NVBP, there was no clear significant trend in NVBP-covered drugs’ DDDc (*P* = .053), while the DDDc of the other tier-one (β_1_ = 0.014, *P* < .001), tier-two (β_1_ = 0.014, *P* < .001), and tier-three (β_1_ = 0.020, *P* < .001) alternative drugs increased gently by months (Table, Figure 3-A/B). Influenced by the pilot NVBP, during the first procurement cycle, the DDDc of NVBP-covered drugs dropped immediately (β_2_ = -3.878, *P* < .001) and kept decreasing over months (β_3_ = -0.068, *P* = .001), which resulted in a 61.55% decrease after half a year. During the second procurement cycle, slight decrease (β_4_ = -0.356, *P* = .049) of NVBP-covered drugs’ DDDc was further observed. For alternative drugs, the increasing trend of DDDc of tier-two alternatives slowed down after the pilot NVBP (β_3_ = -0.029, *P* = .017), but no other significant change was detected.

**Figure 4:**
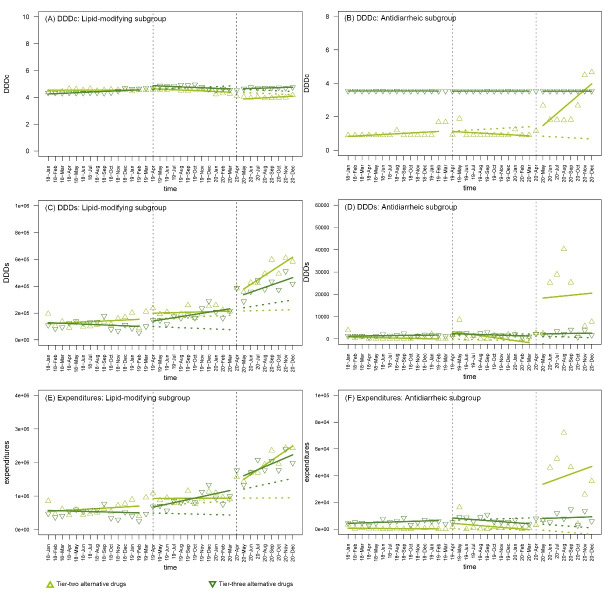
Examples of Subgroup Analyses: Procurement Price, Volume, and Expenditure of the Alternative Drugs from the Lipid-modifying and Antidiarrheic Subgroups, Before and After the Pilot NVBP. Abbreviations: NVBP, National Volume-Based Procurement; DDDs, defined daily doses; DDDc, cost of defined daily dose. Note: 1. The entire findings of the subgroup analyses were available in the supplementary files. 2. Shaded areas represented that data in these month were excluded in this study. Vertical dashed lines represented the initiation of the first/second procurement cycle. 3. Solid lines were fitted based on actual values. Dotted lines simulated the counterfactual scenario where the intervention was not implemented.

 Among the 12 pharmacological subgroups, the magnitude of price reduction of the NVBP-covered drugs varied, which was greater in the lipid-modifying, antihypertensive, antidiarrheic, antiviral subgroups but smaller in the nervous system subgroup (Table S5). For example, the DDDc of the lipid-modifying NVBP-covered drugs dropped immediately upon the initiation of the first (β_2_ = -5.009, *P* < .001) and second (β_4_ = -0.776, *P* < .001) procurement cycles after the pilot NVBP, which resulted in 76.29% and 48.54% reduction in the sixth month after the two interventions, respectively. However, the changes were -24.60% and 0.00% for the psychoanaleptic subgroup (Figure S2, Supplementary file 2). For alternative drugs, unexpected DDDc increases were found in the second procurement cycle for the tier-two alternative drugs in the antidiarrheic (β_5_ = 0.380, P<.001; relative change after half a year: 251.23%) and the anti-inflammatory (β_4_ = 1.359, *P* = .003; relative change after half a year: 42.85%) subgroups (Figure 4B, Figure S3, Table S5 of Supplementary file 3), owing to healthcare institutions beginning to purchase more expensive products with specific INN (Berberine, Ketorolac tromethamine) from new suppliers during this procurement cycle.

**Table:** Effect of the Pilot NVBP on Procurement Price, Volume and Expenditure (Results From ITS Analysis)

**Samples**	**Before the Pilot NVBP**		**In the First Procurement Cycle**			**In the Second Procurement Cycle**		
	**Intercept (β**_0_**)**	**Trend (β**_1_**)**	**Level Change (β**_2_**)**	**Trend Change (β**_3_**)**	**Relative Change**	**Level Change (β**_4_**)**	**Trend Change (β**_5_**)**	**Relative Change**
Procurement price (DDDc, unit: CNY/DDD)								
NVBP-covered drugs	6.297 (*P* < .001)	0.024 (*P* = .053)	-3.878 (*P* < .001)	-0.068 (*P* = .001)	-61.55%	-0.356 (*P* = .049)	0.036 (*P* = .265)	0.00%
Other tier-one alternative drugs	3.290 (*P* < .001)	0.014 (*P* < .001)	0.023 (*P *= .507)	0.005 (*P* = .229)	0.00%	-0.021 (*P* = .607)	-0.003 (*P* = .698)	0.00%
Tier-two alternative drugs	2.836 (*P* < .001)	0.020 (*P* < .001)	0.085 (*P* = .355)	-0.029 (*P* = .017)	-4.47%	0.100 (*P* = .338)	0.000 (P = .981)	0.00%
Tier-three alternative drugs	1.809 (*P* < .001)	0.014 (*P* < .001)	0.079 (*P* = .342)	-0.018 (*P* = .094)	0.00%	-0.010 (*P* = .915)	0.030 (*P* = .094)	0.00%
Procurement volume (DDDs, unit: DDD)								
Bid-winning drugs	1 457 695 (*P* = .293)	75 920 (*P* = .641)	19 227 545 (*P* < .001)	-130 468 (*P *= .581)	1 036.92%	841 095 (*P* = .651)	716 613 (*P* = .046)	20.41%
Non-winning original drugs	4 404 130 (*P* < .001)	189 335 (*P *< .001)	-5 067 460 (*P* < .001)	-173 241 (*P* < .001)	-76.04%	-705 547 (*P* = .066)	30 941 (*P* = .645)	0.00%
Non-winning generic drugs	9 873 582 (*P* < .001)	242 106 (*P* < .001)	-9 993 115 (*P *< .001)	-351 970 (*P* < .001)	-82.71%	-730 794 (*P* = .167)	175 232 (*P* = .070)	0.00%
NVBP-covered drugs	15 185 318 (P < .001)	562 253 (*P *< .001)	2 912 482 (*P* = .009)	-561 213 (*P* = .003)	-3.75%	-1 793 140 (*P* = .204)	726 322 (*P* = .010)	6.27%
Other tier-one alternative drugs	3 206 890 (*P* < .001)	43 717 (*P* = .205)	-346 253 (*P* = .375)	33 423 (*P* = .508)	0.00%	-1 005 911 (*P* = .022)	110 241 (*P* = .150)	0.00%
Tier-two alternative drugs	7 637 906 (*P* < .001)	322 449 (*P* < .001)	-801 254 (*P* = .107)	-35 572 (*P* = .635)	0.00%	-2 096 439 (*P* = .001)	221 127 (*P* = .050)	-8.45%
Tier-three alternative drugs	9 581 230 (*P* < .001)	371 433 (*P *= .007)	-1 246 402 (*P* = .396)	15 421 (*P* = .935)	0.00%	-2 840 424 (*P* = .078)	486 478 (*P* = .094)	0.00%
Procurement expenditure (CNY)								
Bid-winning products	12 328 187 (*P* < .001)	625 959 (*P* = .069)	16 274 624 (*P* < .001)	-1 198 426 (*P* = .021)	106.64%	-3 372 521 (*P* = .410)	1 343 625 (*P* = .077)	0.00%
Non-winning original products	35 376 145 (*P* < .001)	1 323 516 (*P* < .001)	-37 476 183 (*P* < .001)	-1 623 159 (*P* < .001)	-77.70%	-3 817 359 (*P* = .197)	508 739 (*P* = .337)	0.00%
Non-winning generic products	51 340 780 (*P* < .001)	1 648 704 (*P* < .001)	-54 456 900 (*P* < .001)	-2 469 395 (*P* < .001)	-82.16%	-4 978 258 (*P* = .001)	1 246 476 (*P* < .001)	48.15%
NVBP-covered drugs	99 214 277 (*P* < .001)	3 562 972 (*P* < .001)	-72 905 028 (*P* < .001)	-5 624 416 (*P* < .001)	-62.60%	-12 866 086 (*P* = .037)	3 918 726 (*P* < .001)	27.36%
Other tier-one alternative drugs	10 136 873 (*P *< .001)	3 557 345 (*P* < .001)	-1 490 314 (*P* = .092)	135 120 (*P* = .298)	0.00%	-3 944 823 (*P* = .001)	394 900 (*P* = .042)	4.38%
Tier-two alternative drugs	22 136 025 (*P *< .001)	1 116 878 (*P* < .001)	488 512 (*P* = .872)	-580 384 (*P* = .149)	0.00%	-4 293 581 (*P* = .194)	1 230 507 (*P* = .044)	10.28%
Tier-three alternative drugs	16 070 538 (*P* < .001)	1 023 580 (*P* = .001)	-1 656 692 (*P* = .585)	-422 418 (*P* = .288)	0.00%	-5 045 630 (*P* = .129)	1 774 530 (*P* = .005)	8.86%

Abbreviations: NVBP, National Volume-Based Procurement; ITS, interrupted time series; DDDs, defined daily doses; DDDc, cost of defined daily dose. *Note*: 1. NVBP-covered drugs included both the bid-winning and non-winning drugs. 2. Relative change in this table refers to relative change at the sixth month after the intervention.

###  Changes in Procurement Volume (DDDs)

 Descriptive analyses showed that (Table S4), before the pilot NVBP, non-winning drugs comprised a large part of the total procurement volumes of the NVBP-covered drugs, much higher than the bid-winning drugs (non-winning versus bid-winning: 89.41% versus 10.59% from May to December 2018). Conversely, the bid-winning drugs became dominant after the policy (non-winning versus bid-winning: 19.23% versus 80.77% from May to December 2019; 13.87% versus 86.13% from May to December 2020).

 According to the ITS analyses, the DDDs of bid-winning drugs increased dramatically and instantly after the pilot NVBP in the first procurement cycle (β_2_ = 1.92×10^7^, *P* < .001; relative change after half a year: 1036.91%) and kept rising during the second procurement cycle (β_5_ = 7.17×10^5^, *P* = .046; relative change after half a year: 20.41%). Meanwhile, the procurement volume of non-winning generic and original drugs both decreased during the first procurement cycle (relative change after half a year: -82.71% versus -76.04%). For NVBP-covered drugs, the increase of DDDs (β_1_ = 5.62×10^5^, *P* < .001) slowed down and remained stable after the pilot NVBP (β_3_ = -5.61×10^5^, *P* = .003). No significant increase in the other tire-one, tier-two and tier-three alternatives was observed after the policy (Table, Figure 3-C/D).

 Although no significant increase was found for the overall DDDs of NVBP-covered drugs, the DDDs of NVBP-covered drugs in the subgroups of lipid-modifying and antivirals drugs rose significantly after that the pilot NVBP, possibly indicating the release of medication demand (Table S6, Supplementary file 4). Conversely, NVBP-covered drugs for obstructive airway diseases were observed with both level (β_2_ = -1.85×10^5^, *P* = .001) and trend (β_3_ = -3.10×10^4^, *P* < .001) decline in DDDs after the pilot NVBP, which could be partially explained by the influence of COVID-19. For the psychoanaleptic subgroup, there was also a decrease in NVBP-covered drugs’ DDDs after the pilot NVBP, with no significant change in bid-winning drugs’ DDDs. The reason is possibly that NVBP-covered drugs in this subgroup had the minimal price reduction as mentioned above. For non-winning original and generic drugs in subgroups, the antineoplastic non-winning original drugs were the exceptions, as no significant decrease in DDDs was observed during the two procurement cycles after the pilot NVBP (β_2_ = 405, *P* = .708; β_3_ = -197, *P* = .169; β_4_ = 1.60×10^3^, *P* = .176; β_5_ = 148, *P* = .482) (Figure S4).

 Notably, there were significant increases in the procurement volume of alternative drugs in the antihypertensive, lipid-modifying, psychoanaleptic, antiepileptic, antiviral, antibacterial, antidiarrheic, and anti-inflammatory/antirheumatic subgroups (Figure 4-C/D, Figure S5). This suggested the undesirable “spillover” effect of the policy, which may result from the additional financial incentives provided to healthcare institutions to purchase alternative drugs, as their transaction process may be less transparent. Moreover, the increase in procurement volume and price of the antidiarrheic tier-two alternatives (β_4_ = 2.30×10^5^, *P* < .001; β_5_ = -225, *P* = .795) indicated healthcare institutions not only purchased more alternative drugs but also preferred alternative products with higher prices (Table S6). Furthermore, there was no clear correlation discovered between the increase in alternative drugs’ procurement volume and pharmacological characteristics (subgroup types) or tiers of substitutability for the bid-winning drugs.

###  Changes in Procurement Expenditure

 Similar as the descriptive results about DDDs (Table S4), non-winning drugs contributed most of NVBP-covered drugs’ procurement expenditure before the pilot NVBP (non-winning versus bid-winning: 86.12% versus 13.88% in May to December 2018), but after the policy, bid-winning drugs’ procurement expenditure dramatically increased, accounting for more than half of the total (non-winning versus bid-winning: 47.87% versus 52.13% from May to December 2019; 41.91% versus 58.09% from May to December 2020).

 After the pilot NVBP, during the first procurement cycle, the procurement expenditure of NVBP-covered drugs decreased significantly (β_2_ = -7.29×10^7^, *P* < .001; β_3_ = -5.62×10^6^, *P* < .001; relative change after half a year: -62.6%), revealing the cost-saving effects of the NVBP, while this trend moderated in the second procurement cycle. However, this cost-saving effect may be weakened as the procurement expenditure of the alternative drugs significantly increased (Table, Figure 3-E/F), during the second procurement cycle after the pilot NVBP (relative change after half a year: other tier-one: 4.38%, tier-two: 10.28%, tier-three: 8.86%).

 The cost-saving effects of the pilot NVBP on NVBP-covered drugs has been found in most subgroups and with varying degrees, except the antineoplastic subgroup (Table S7 of Supplementary file 5, Figure S6). Among the 12 subgroups, most of the bid-winning drugs’ procurement expenditure increased slightly, aside from the psychoanaleptics and the anti-inflammatory/antirheumatic subgroups since their procurement volume did not increase significantly as other subgroups.

 Similar to the findings from the procurement volume, during the second procurement cycle, in the subgroups of antidiarrheic, antibacterial, lipid-modifying and antiviral drugs, the procurement expenditure of their alternative drugs increased along with the volumes as mentioned above (Table S7, Figure S7).

## Discussion

 The current study find that the pilot NVBP implemented in Tianjin lowered the procurement price of NVBP-covered drugs, improved the market concentration by promoting the substitution of bid-winning drugs for non-winning drugs, and was cost-saving, in both the first and second procurement cycle. However, in some pharmacological subgroups, the procurement volume and expenditure of alternative drugs grew during the second procurement cycle, which may diminish the overall benefits of the policy.

 Experience from many international organizations and other countries have demonstrated that CDP can effectively reduce drug prices,^6-13^ with reductions ranging from -52% to -2.1%.^6,9^ China’s NVBP policy has shown a greater potential for lowering drug price, which may benefit from the greater purchasing power created by China’s enormous market size. In this study, the procurement prices of the NVBP-covered drugs decreased by 61.55% during the first procurement cycle of the pilot NVBP and did not rebound in the second procurement cycle. This price reduction effects of the pilot NVBP have also been observed by previous studies conducted in Shenzhen, the other pilot city.^16,17,26^ Moreover, Yuan have found the price reductions of bid-winning drugs varied from 21% to 96% after the pilot NVBP, when comparing their bid-winning prices with the their lowest price in the pilot cities one year before the policy.^4^

 The procurement volume increased for bid-winning drugs, while decreased for non-winning drugs, which was consistent with the findings from previous studies on China’s NVBP,^15-17,27^ but few studies on the CDP conducted in other countries have paid attention to this research question. As mentioned before, China has highly fragmented drug manufacturing sectors,^14^ thus it is not uncommon for many manufactures to produce drugs with the same INN. The NVBP policy marked a turning point, and by augmenting the market share of the bid-winning drugs, it improved market concentration. Moreover, considering 23 out of 25 bid-winning drugs in the pilot NVBP were generics, this policy tremendously promoted the generic substitution, which is known as an effective way to improve drug affordability and accessibility.^4,15,28^

 Significant increases in the NVBP-covered drugs’ procurement volume were observed in antiviral and lipid-modifying subgroups. Taking antiviral subgroup as an example, six months after the pilot NVBP, the DDDs of NVBP-covered drugs (Entecavir, Tenofovir disoproxil) increased by 40.70%, which, however, was lower than the descriptive pre- and post-policy growth rate (95.60%) reported in the study based on another pilot city, Shenzhen.^16^ Aside from the difference in study location and study period, the difference in the growth rate could be partially explained as the relative change (40.70%) in this study was calculated based on ITS regression model so that the pre-intervention trend of DDDs was controlled. Previous studies indicated that only 11% of patients with hepatitis B disease received standardized antiviral treatment in China in 2016, due to the poor drug affordability.^16,29,30^ After the pilot NVBP, the DDDc of antiviral NVBP-covered drugs (Entecavir and Tenofovir disoproxil) dropped by 76.33% within six months, providing an opportunity to release patients’ demand for them.

 In 7 out of 12 pharmacological subgroups, the procurement volume and expenditure of alternative drugs (tier-two or tier-three) increased significantly after the pilot NVBP, mainly during the second procurement cycle. Research on the CDP conducted in other countries rarely focuses on the alternative drugs, while above undesired “spillover” effect was also detected by previous studies in Shenzhen, China after the pilot NVBP, for antibacterial drugs, but not for antihypertensive or antiviral drugs.^16-18^ A possible explanation could be the extra financial incentives for healthcare institutions to purchase alternative drugs that have less transparent transaction process. To be more specific, in NVBP, bid-winning drugs’ manufactures no longer need to conduct market research, negotiate with individual healthcare institutions, or promote their products with competition from other manufactures.^7,14,31^ However, these process might still be necessary for promoting the alternative drugs, and the related administration and transaction cost may partly been transformed into the financial incentives to healthcare institutions. Alternative drugs in the antidiarrheic and the anti-inflammatory/antirheumatic subgroups, berberine and ketorolac tromethamine, were interesting examples. During the second procurement cycle after the pilot NVBP, healthcare institutions began to purchase these drugs from new manufactures with the unit sale price up to 6 times higher than before, and their procurement volume also increased significantly by month. It was worth noting that the majority of these undesirable “spillover” effects on alternative drugs were observed in the second procurement cycle after the policy. This might be attributed to the fact that, after the first procurement cycle, healthcare institutions got familiar with the NVBP rules and might become more manipulative when seeking financial incentives.

 Although the procurement volume of bid-winning drugs increased dramatically after the pilot NVBP, more than CNY 550 million was saved for purchasing NVBP-covered drugs within 6 months after the NVBP in Tianjin based on the ITS results. According to official reports, the accumulated cost saving from NVBP over the past 3 years reached a total of CNY 260 billion nationwide.

 This study provides new evidence that expands public’s understanding of the impacts of the NVBP, especially about its effects on alternative drugs, and the diverse effects across different procurement cycles and pharmacological categories. The study design is rigorous thus the possible effects of the other policies are largely eliminated.

 This study also has several limitations. First, upon meticulously reviewing the policies and events occurred over the three-year study period, it was found that uncontrollable confounding factors still persisted, potentially introducing bias to the results, which included the policy to reduce the tax and price of antineoplastic drugs that carried out in Tianjin in December 2018, the update of the Chinese guideline of prevention and treatment for chronic hepatitis B in December 2019,^29^ and the outbreak of COVID-19, as well as the periodic price adjustment conducted by procurement platform in Tianjin at the end of 2019. These confounding effects can be observed in some pharmacological subgroups, mainly in antineoplastic and antiviral subgroups and drugs for obstructive airway diseases, suggesting careful interpretations of findings were needed. Nevertheless, for the main analysis and the rest of subgroups, the findings were consistent and robust. Second, single-group ITS analysis was conducted rather than ITS analysis with a control group because of the limited data accessibility to non-pilot cities. Third, the alternative drugs were defined in accordance with the recommendations of the NHSA, taking into account chemical characteristics of drugs, and regulatory and clinical perspectives. However, not all drugs treating the same diseases were included, and it is recommended to conduct further exploration on the definition of alternative drugs in relation to different purposes. Forth, the generalizability of the study results is restricted as the impact of NVBP was only explored in single provincial city, although Tianjin was one of the cities piloting the NVBP firstly in China. Moreover, further investigation into the impact of NVBP on drug utilization, clinical benefit, and economic burden, using individual-level data, is warranted.

## Conclusion

 The pilot NVBP policy in China is effective in reducing the procurement price, promoting generic substitution, and saving procurement expenditure. However, the increase in procurement price, volume and expenditure of the alternative drugs across various pharmacological categories reveals the significance of regulating healthcare institutions’ drug purchasing behavior, particularly for drugs that not covered by NVBP but are substitutable for bid-winning drugs in clinical practice.

## Ethical issues

 This study used secondary drug procurement data from Tianjin Medical Purchasing Center. As such, ethical approval was not required.

## Competing interests

 Authors declare that they have no competing interests.

## Funding

 This work was supported by the Tianjin Medical Purchasing Center of Tianjin Municipal Medical Insurance Bureau in China through a commissioned research project (grant number: Not applicable). The funder provided the data used of the current study, and the funder had no role in study design, data analysis, preparation and review of the manuscript.

## Data availability statement

 The data that support the findings of this study are available from Tianjin Medical Purchasing Center of Tianjin Municipal Medical Insurance Bureau but restrictions apply to the availability of these data, which were used under license for the current study, and so are not publicly available. Data are however available from the authors upon reasonable request and with permission of Tianjin Municipal Medical Insurance Bureau.

## Disclaimers

 The views expressed in this article are for the authors and not the position of the institutions of affiliation or the funder.

## Supplementary files


Supplementary file 1. Supplementary Figures and Tables About the Timeline of Policies, Details of the Study Samples, and Results of Descriptive Analysis (Figure S1, Table S1 to S4).

Supplementary file 2. Supplementary Figures of Subgroup Analysis Results (Figure S2-S7).

Supplementary file 3. Supplementary Table of the Subgroup Analysis Results for Procurement Price (Table S5).

Supplementary file 4. Supplementary Table of the Subgroup Analysis Results for Procurement Volume (Table S6).

Supplementary file 5. Supplementary Table of the Subgroup Analysis Results for Procurement Expenditure (Table S7).
