# Supplementary material for: Impact of China’s National Volume-Based Procurement on Drug Procurement Price, Volume, and Expenditure: An Interrupted Time Series Analysis in Tianjin
Source: Int J Health Policy Manag. 2023 Sep 20;12:7724. doi: 10.34172/ijhpm.2023.7724 (PMC10590231; doi:10.34172/ijhpm.2023.7724)
Supplement: Supplementary file 1 — Supplementary Figures and Tables About the Timeline of Policies, Details of the Study Samples, and Results of Descriptive Analysis (Figure S1, Table S1 to S4). [file ijhpm-12-7724-s001.pdf]

Article title: Impact of China's National Volume-Based Procurement on Drug Procurement Price, Volume, and Expenditure: An Interrupted Time Series Analysis in Tianjin

Journal name: International Journal of Health Policy and Management (IJHPM)

Authors' information: Boya Zhao[1,2], Jing Wu[1,2]*

[1]School of Pharmaceutical Science and Technology, Tianjin University, Tianjin, China.

[2]Center for Social Science Survey and Data, Tianjin University, Tianjin, China.

***Correspondence to**: Jing Wu, Email: jingwu@tju.edu.cn

Citation: Zhao B, Wu J. Impact of China's national volume-based procurement on drug procurement price, volume, and expenditure: an interrupted time series analysis in Tianjin. Int J Health Policy Manag. 2023;12:7724. doi:10.34172/ijhpm.2023.7724

Supplementary file 1. Supplementary Figures and Tables About the Timeline of Policies, Details of the Study Samples, and Results of Descriptive Analysis (Figure S1, Table S1 to S4).

Main Content:

Figure S1 (Page 2). The timeline of NVBP policies

Table S1 (Page 3-6). Information of the bid-winning products in the pilot NVBP in Tianjin

Table S2 (Page 7-8). Alternative drugs for the bid-winning drugs in the pilot NVBP in the current study

Table S3 (Page 9). Drugs in different pharmacological groups in subgroup analysis

Table S4 (Page 10). Descriptive analysis of the monthly average procurement price, volume, and expenditure before the pilot NVBP and during the first and second procurement cycles after the pilot NVBP

Note: The testing results of autocorrelation and stationary series are available by contacting the authors.

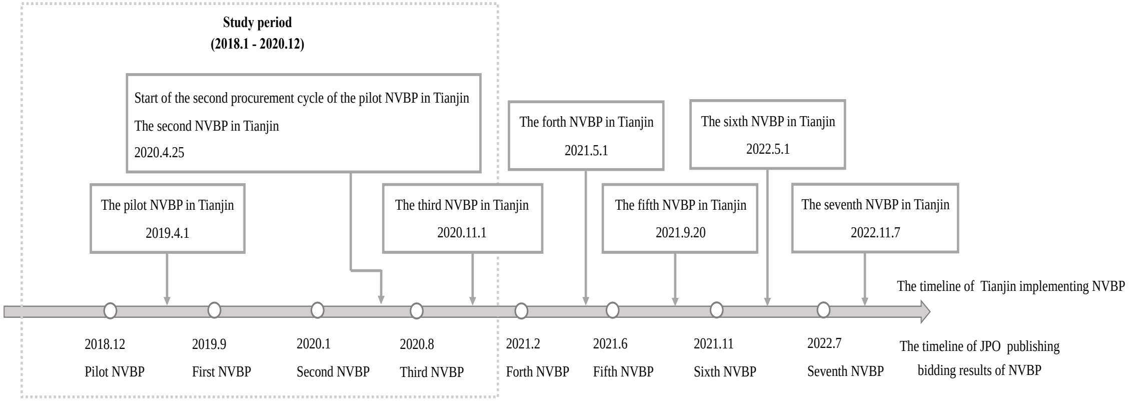

Figure S1. The timeline of NVBP policies

Note:

1. The First NVBP was also be called “Pilot NVBP Expansion” because it tendered the same drugs as the pilot NVBP but were implemented in other areas in China aside from the 11 pilot cities.

2. Only the start date of each NVBP’s first procurement cycle is displayed in this Figure, except for the pilot NVBP that the start date of its second procurement cycle is also shown.

3. NVBP is short for “National Volume-based Procurement”. JPO is short for “Joint Procurement Office”.

Table S1. Information of the bid-winning drugs in the pilot NVBP in Tianjin

	In the First Procurement Cycle						In the Second Procurement Cycle					
**INN**	**Specification**	**Form**	**Package size**	**Manufacturer**	**Bid-winning price (CNY/package)**	**Original**	**Specification**	**Form**	**Package size**	**Manufacturer**	**Bid-winning price (CNY/package)**	**Original**
**Atorvastatin**												
	10mg	Table	7	Beijing Jialin	3.88	No	10mg	Tablet	14	Lepu	4.41	No
	20mg	Table	7	Beijing Jialin	6.60	No	10mg	Tablet	14	Qilu	1.68	No
						No	20mg	Tablet	7	Lepu	3.84	No
						No	20mg	Tablet	14	Qilu	2.86	No
**Rosuvastatin**												
	5mg	Tablet	28	Zhejiang Jingxin	12.82	No	*	*	*	*	3.95	*
	10mg	Tablet	28	Zhejiang Jingxin	21.80	No	*	*	*	*	6.71	*
**Amlodipine**												
	5mg	Tablet	28	Zhejiang Jingxin	4.16	No	*	*	*	*	1.57	*
**Enalapril**												
	5mg	Tablet	16	Yangtze River	5.25	No	*	*	*	*	*	*
	10mg	Tablet	16	Yangtze River	8.93	No	*	*	*	*	*	*
**Lisinopril**												
	10mg	Tablet	28	Zhejiang Huahai	6.45	No	*	*	*	*	*	*
**Fosinopril**												

	10mg	Tablet	14	Sino-American Shanghai Squibb	11.80	Yes	*	*	*	*	*	*
**Losartan**												
	50mg	Tablet	7	Zhejiang Huahai	7.54	No	50mg	Tablet	28	Zhejiang Huahai	28.67	No
	50mg	Tablet	14	Zhejiang Huahai	14.70	No	*	*	*	*	*	*
	100mg	Tablet	7	Zhejiang Huahai	12.82	No	*	*	*	*	*	*
	100mg	Tablet	14	Zhejiang Huahai	24.99	No	*	*	*	*	*	*
**Irbesartan**												
	75mg	Tablet	28	Zhejiang Huahai	5.66	No	*	*	*	*	5.20	*
	75mg	Tablet	42	Zhejiang Huahai	8.37	No	*	*	*	*	7.69	*
**Irbesartan and hydrochlorothiazide**												
	150mg/12.5mg	Tablet	14	Zhejiang Huahai	15.26	No	*	*	*	*	14.28	*
	150mg/12.5mg	Tablet	28	Zhejiang Huahai	29.76	No	*	*	*	*	27.85	*
**Olanzapine**												
	5mg	Tablet	14	Jiangsu Hanson	77.44	No	*	*	*	*	50.01	*
	10mg	Tablet	7	Jiangsu Hanson	67.51	No	*	*	*	*	43.60	*
**Risperidone**												
	1mg	Tablet	60	Zhejiang Huahai	10.02	No	*	*	*	*	7.20	*
**Dexmedetomidine**												
	2ml:0.2mg	Injection	4	Yangtze River	532.00	No	*	*	*	*	*	*
**Paroxetine**												
	20mg	Tablet	14	Zhejiang Huahai	23.69	No	*	*	*	*	22.11	*
	20mg	Tablet	20	Zhejiang Huahai	33.40	No	*	*	*	*	31.18	*

**Escitalopram**												
	10mg	Tablet	7	Sichuan Kelun	30.94	No	*	*	*	*	27.86	*
	10mg	Tablet	10	Sichuan Kelun	43.63	No	*	*	*	*	39.28	*
	10mg	Tablet	14	Sichuan Kelun	60.33	No	*	*	*	*	54.33	*
**Levetiracetam**												
	250mg	Tablet	30	Zhejiang Jingxin	72.00	No	*	*	*	*	71.79	*
**Gefitinib**												
	250mg	Tablet	10	AstraZeneca	547.00	Yes	*	*	*	*	*	*
**Imatinib**												
	100mg	Tablet	60	Jiangsu Hanson	623.82	No	*	*	*	*	586.00	*
**Pemetrexed**												
	100mg	Injection	1	Sichuan Huiyu	810.00	No	*	*	*	*	798.00	*
	500mg	Injection	1	Sichuan Huiyu	2776.97	No	*	*	*	*	2735.83	*
**Tenofovir disoproxil**												
	300mg	Tablet	10	Chendu Brilliant	6.15	No	*	*	*	*	4.85	*
	300mg	Tablet	30	Chendu Brilliant	17.72	No	*	*	*	*	13.98	*
**Entecavir**												
	0.5mg	Tablet	28	Chiatai Tianqing	17.36	No	0.5mg	Tablet	21	Suzhou Dawnrays	3.83	No
**Cefuroxime**												
	250mg	Tablet	6	Chendu Brilliant	3.16	No	*	*	*	*	2.69	*
	250mg	Tablet	12	Chendu Brilliant	6.16	No	*	*	*	*	5.26	*
**Clopidogrel**												
	25mg	Tablet	10	Shenzhen Salubris	13.54	No	*	*	*	*	10.64	*
	25mg	Tablet	20	Shenzhen Salubris	26.40	No	*	*	*	*	20.75	*

	75mg	Tablet	7	Shenzhen Salubris	22.26	No	*	*	*	*	17.50	*
**Flurbiprofen axetil**												
	5ml:50mg	Injection	5	Beijing Tide	109.75	No	*	*	*	*	109.40	*
**Montelukast**												
	10mg	Tablet	5	Shanghai Anbison	19.38	No	*	*	*	*	18.96	*
**Montmorillonite**												
	3g	Powder	10	Hainan Slmcere	6.80	No	3g	Powder	12	Harbin Pharmaceutical	3.60	*
	3g	Powder	15	Hainan Slmcere	10.20	No	3g	Powder	12	Zhejiang Hailisheng	3.95	*
							3g	Powder	15	Hunan Warrant	4.16	*

Note: 1. "*"indicated that the drug information in the second procurement cycle in this cell was exactly as same as in the first procurement cycle in the same line. 2. NVBP is short for "National Volume-based Procurement".

Table S2. Alternative Drugs for the Bid-winning Drugs in the pilot NVBP in the Current Study

INN of the bid-winning drugs (Administration route)	Alternative drugs		
	Tier-one	Tier-two	Tier-three
Atorvastatin (Oral)	NWDP	Pravastatin, Fluvastatin, Lovastatin	XueZhiKang, ZhiBiTuo, ZhiBiTai
Rosuvastatin (Oral)	NWDP	[Same as "Atorvastatin (Oral)"]	[Same as "Atorvastatin (Oral)"]
Irbesartan (Oral)	NWDP	Telmisartan	Allisartan medoxomil, Olmesartan medoxomil/Amlodipine, Valsartan/Amlodipine, Olmesartan medoxomil/Hydrochlorothiazide, Losartan potassium/Hydrochlorothiazide, Telmisartan/Hydrochlorothiazide, Valsartan/Hydrochlorothiazide, Candesartan cilexetil/Hydrochlorothiazide
Losartan (Oral)	NWDP	[Same as "Irbesartan (Oral)"]	[Same as "Irbesartan (Oral)"]
Amlodipine (Oral)	NWDP, Levamlodipine, Amlodipine/Folic acid	Felodipine, Nifedipine	Amlodipine/Atorvastatin, Amlodipine/Benazepril, Olmesartan medoxomil/Amlodipine, Valsartan/Amlodipine
Irbesartan/Hydrochlorothiazide (Oral)	NWDP	Olmesartan medoxomil/Hydrochlorothiazide, Losartan potassium/Hydrochlorothiazide, Telmisartan/Hydrochlorothiazide, Valsartan/Hydrochlorothiazide, Candesartan cilexetil/Hydrochlorothiazide	Telmisartan, Allisartan Medoxomil, Olmesartan medoxomil/Amlodipine, Valsartan/Amlodipine
Fosinopril (Oral)	NWDP	Benazepril, Ramipril, Perindopril	Amlodipine/Benazepril, Benazepril/Hydrochlorothiazide, Lisinopril/Hydrochlorothiazide, Enalapril/Folic acid, Perindopril/Indapamide, Perindopril/Amlodipine
Lisinopril (Oral)	NWDP	[Same as "Fosinopril (Oral)"]	[Same as "Fosinopril (Oral)"]
Enalapril (Oral)	NWDP	[Same as "Fosinopril (Oral)"]	[Same as "Fosinopril (Oral)"]
Olanzapine (Oral)	NWDP	Paliperidone	Aripiprazole, Amisulpride, Haloperidol, Chlorpromazine, Ziprasidone

Risperidone (Oral)	NWDP	Quetiapine	Aripiprazole, Amisulpride, Haloperidol, Chlorpromazine, Ziprasidone, Perphenazine, Fluphenazine decanoate, Paliperidone, Sulpiride, Penfluridol, Tiapride
Dexmedetomidine (Injectable)	NWDP	Midazolam	
Escitalopram (Oral)	NWDP		Vortioxetine, Duloxetine, Fluvoxamine, Bupropion, Trazodone
Paroxetine (Oral)	NWDP	Vortioxetine, Duloxetine	Fluvoxamine, Bupropion, Trazodone
Levetiracetam (Oral)	NWDP		Oxcarbazepine, Magnesium Valproate, Sodium Valproate, Carbamazepine, Lamotrigine, Topiramate
Imatinib (Oral)	NWDP		Dasatinib
Gefitinib (Oral)	NWDP	Icotinib, Erlotinib, Afatinib	
Pemetrexed (Injectable)	NWDP		
Entecavir (Oral)	NWDP	Tenofovir alafenamide	Lamivudine, Telbivudine
Tenofovir disoproxil (Oral)	NWDP, Tenofovir alafenamide		[Same as “Entecavir (Oral)”]
Cefuroxime (Oral)	NWDP		Cefalexin, Cefprozil, Cefdinir, Cefixime, Cefadroxil
Clopidogrel (Oral)	NWDP		Aspirin, Ticlopidine, Cilostazol
Montelukast (Oral)	NWDP		
Flurbiprofen axetil (Injectable)	NWDP	Parecoxib, Ketorolac tromethamine	
Montmorillonite (Oral)	NWDP	Berberine	Loperamide, Tannalbin

Note:

1. Alternative drugs for the bid-winning drugs were defined in accordance with the guidance of an official document “Monitoring Plan Work of National Centralized Drug Procurement and Use” issued by the National Healthcare Security Administration (NHSA) and released in June 2020.

2. All alternative drugs’ administration routes (injectable or oral) are as same as the corresponding bid-winning drug drugs.

3. Some alternative drugs recommended by the “Monitoring Plan Work of National Centralized Drug Procurement and Use” were not included in this table to avoid confounding effects of other policies during the study period, which included alternative drugs that have the same International Nonproprietary Names (INNs) as bid-winning drugs in the pilot (except non-winning drugs) or second or third NVBP, and antineoplastic drugs that were newly added to the National Reimbursement Drug List through pricing negotiation during study period.

4. XueZhiKang, ZhiBiTuo and ZhiBiTai are Chinese traditional medicines.

5. NVBP is short for “National Volume-based Procurement”. NWDP is short for “non-winning products”. INN is short for “International Nonproprietary Names”.

Table S3. Drugs in different pharmacological groups in subgroup analysis

**Anatomical main group**	**Pharmacological subgroup**	**Numbers**	**INN of Bid-winning drugs**
Cardiovascular	Antihypertensives	7	Irbesartan, Losartan, Amlodipine, Fosinopril, Lisinopril, Enalapril, Irbesartan/Hydrochlorothiazide
	Lipid-modifying drugs	2	Atorvastatin, Rosuvastatin
Nervous	Psycholeptics	3	Olanzapine, Risperidone, Dexmedetomidine
	Psychoanaleptics	2	Escitalopram, Paroxetine
	Antiepileptics	1	Levetiracetam
Antineoplastic	Antineoplastics	3	Imatinib, Gefitinib, Pemetrexed
Antiinfectives	Antivirals	2	Entecavir, Tenofovir disoproxil
	Antibacterials	1	Cefuroxime
Blood and blood forming organs	Antithrombotics	1	Clopidogrel
Alimentary tract and metabolism	Antidiarrheics	1	Montmorillonite
Musculo-skeletal	Anti-inflammatory/antirheumatic drugs	1	Flurbiprofen axetil
Respiratory	Drugs for obstructive airway diseases	1	Montelukast

Table S4. Descriptive analysis of the monthly average procurement price, volume, and expenditure before the pilot NVBP and during the first and second procurement cycles after the pilot NVBP

	Procurement price (DDDc, unit: CNY/DDD)			Procurement volume (DDDs, unit: DDD)			Procurement expenditure (CNY)		
	May.to Dec. 2018	May.to Dec. 2019	May.to Dec. 2020	May.to Dec. 2018	May.to Dec. 2019	May.to Dec. 2020	May.to Dec. 2018	May.to Dec. 2019	May.to Dec. 2020
	Monthly average values			Monthly average values			Monthly average values		
Bid-winning drugs	——	——	——	2 126 696	21 389 183	25 242 863	17 898 459	35 505 022	31 846 249
Non-winning original drugs	——	——	——	5 870 039	2 158 708	2 065 252	45 346 041	15 738 804	12 604 074
Non-winning generic drugs	——	——	——	12 087 394	2 932 736	1 999 159	65 700 786	16 866 828	10 369 273
NVBP-covered drugs	6.42	2.58	1.88	20 080 721	26 477 176	29 303 760	128 945 284	68 110 654	54 819 596
Other tier-one alternative drugs	3.41	3.63	3.82	3 532 413	4 323 098	3 869 418	12 031 487	14 023 500	16 552 012
Tier-two alternative drugs	2.98	3.20	3.21	10 351 818	13 244 858	15 807 560	30 798 187	42 242 660	50 590 439
Tier-three alternative drugs	1.90	2.08	2.18	12 605 730	16 120 769	20 009 585	24 018 063	33 526 685	43 650 060

Note: 1. May. To Dec. 2018: study period before the pilot NVBP, for descriptive analysis; May. To Dec. 2019: study period during the first procurement cycle after the pilot NVBP, for descriptive analysis. May. To Dec. 2020: study period during the second procurement cycle after the pilot NVBP, for descriptive analysis.

2. NVBP is short for "National Volume-based Procurement"

3. The DDDc of both the bid-winning and non-winning drugs were not reported as some of them were not purchased in some months, resulting in the inability to calculate their DDDc values.
