# Supplementary material for: Impact of China’s National Volume-Based Procurement on Drug Procurement Price, Volume, and Expenditure: An Interrupted Time Series Analysis in Tianjin
Source: Int J Health Policy Manag. 2023 Sep 20;12:7724. doi: 10.34172/ijhpm.2023.7724 (PMC10590231; doi:10.34172/ijhpm.2023.7724)
Supplement: Supplementary file 2 — Supplementary Figures of Subgroup Analysis Results (Figure S2-S7). [file ijhpm-12-7724-s002.pdf]

Article title: Impact of China's National Volume-Based Procurement on Drug Procurement Price, Volume, and Expenditure: An Interrupted Time Series Analysis in Tianjin

Journal name: International Journal of Health Policy and Management (IJHPM)

Authors' information: Boya Zhao[1,2], Jing Wu[1,2]*

[1]School of Pharmaceutical Science and Technology, Tianjin University, Tianjin, China.

[2]Center for Social Science Survey and Data, Tianjin University, Tianjin, China.

***Correspondence to**: Jing Wu, Email: jingwu@tju.edu.cn

Citation: Zhao B, Wu J. Impact of China's national volume-based procurement on drug procurement price, volume, and expenditure: an interrupted time series analysis in Tianjin. Int J Health Policy Manag. 2023;12:7724. doi:10.34172/ijhpm.2023.7724

Supplementary file 2. Supplementary Figures of Subgroup Analysis Results (Figure S2-S7).

Main Content:

Figure S2 (Page 2). Subgroup Analysis: Procurement Price of NVBP-covered Drugs before and after the Pilot NVBP

Figure S3 (Page 3). Subgroup Analysis: Procurement Price of Alternative Drugs before and after the Pilot NVBP

Figure S4 (Page 4). Subgroup Analysis: Procurement Volume of Bid-winning, Non-winning, and NVBP covered Drugs before and after the Pilot NVBP

Figure S5 (Page 5). Subgroup Analysis: Procurement Volume of Alternative Drugs before and after the Pilot NVBP

Figure S6 (Page 6). Subgroup Analysis: Procurement Expenditure of Bid-winning, Non-winning, and NVBP covered Drugs before and after the Pilot NVBP

Figure S7 (Page 7). Subgroup Analysis: Procurement Expenditure of Alternative Drugs before and after the Pilot NVBP

Note: The testing results of autocorrelation and stationary series are available by contacting the authors.

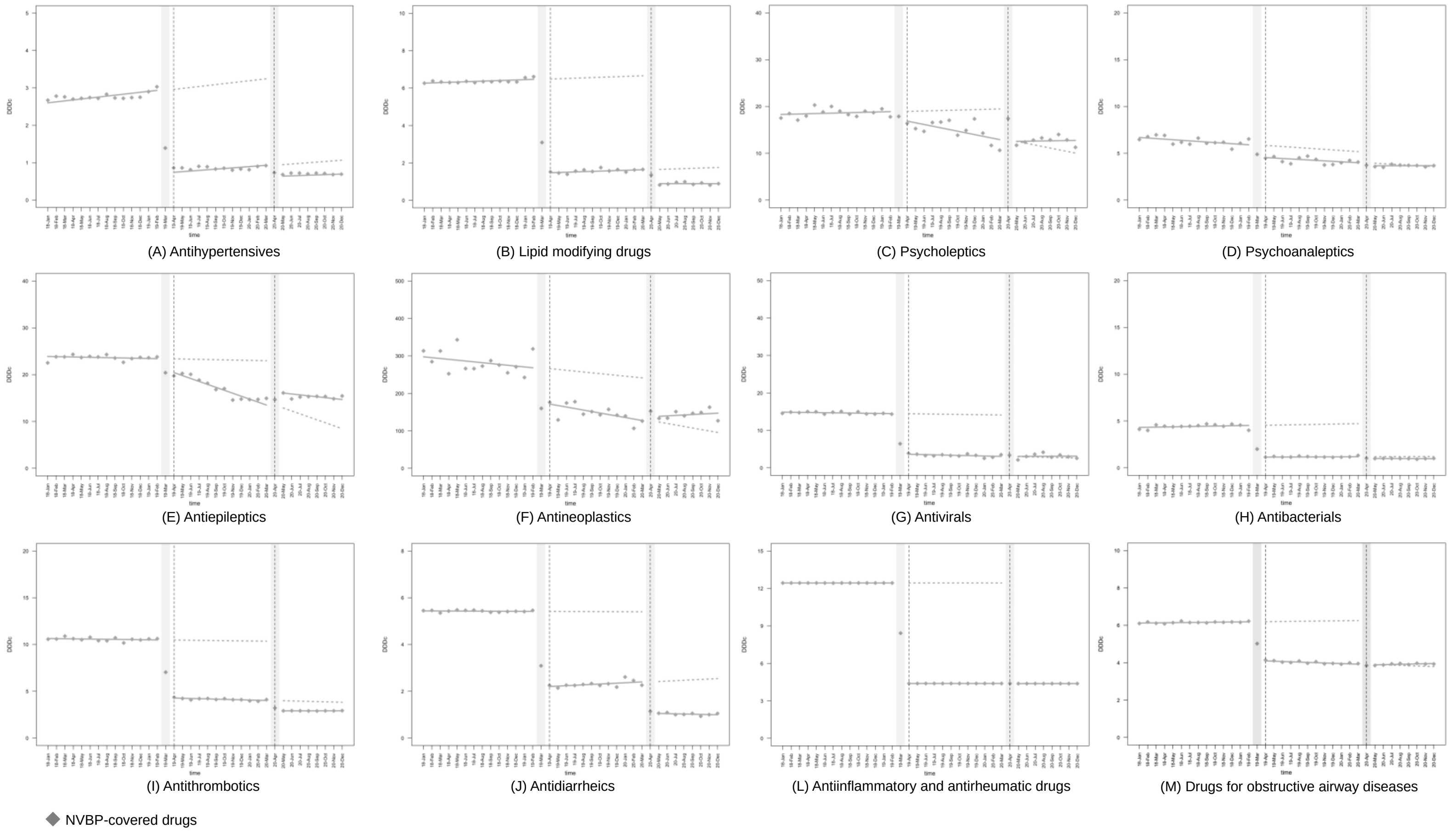

Figure S2. Subgroup Analyses: Procurement Price of NVBP-covered Drugs before and after the Pilot NVBP

Note: 1. Shaded area represents that data in this month was excluded in this study. Vertical dashed line represents that the intervention was implemented in this month. 2. Solid line is fitted based on actual values. Dotted line simulates the counterfactual scenario where the intervention was not implemented.3. NVBP is short for “National Volume-based Procurement”.

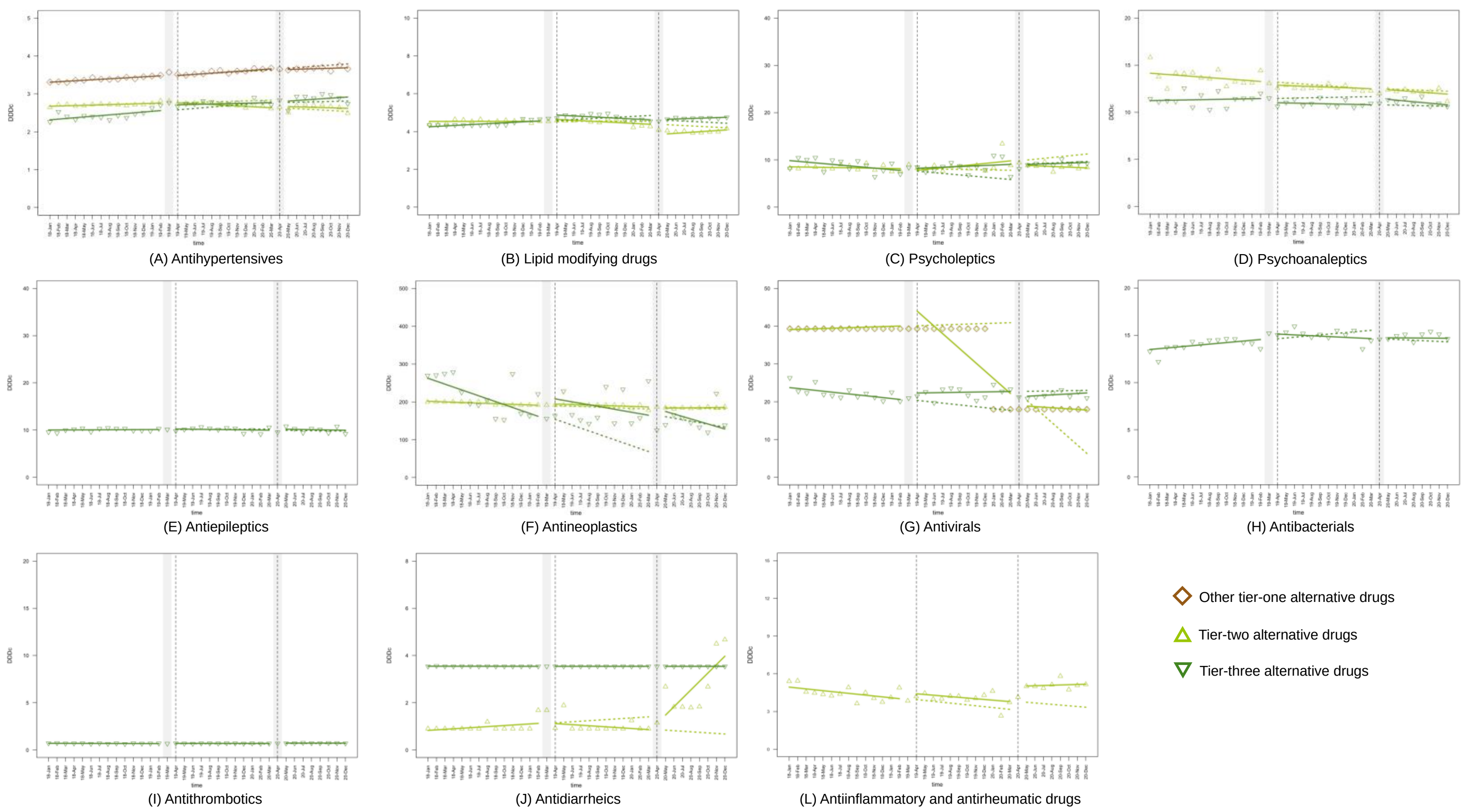

Figure S3. Subgroup Analyses: Procurement Price of Alternative Drugs before and after the Pilot NVBP

Note:1. Shaded area represents that data in this month was excluded in this study. Vertical dashed line represents that the intervention was implemented in this month. 2. Solid line is fitted based on actual values. Dotted line simulates the counterfactual scenario where the intervention was not implemented. 3. NVBP is short for "National Volume-based Procurement".

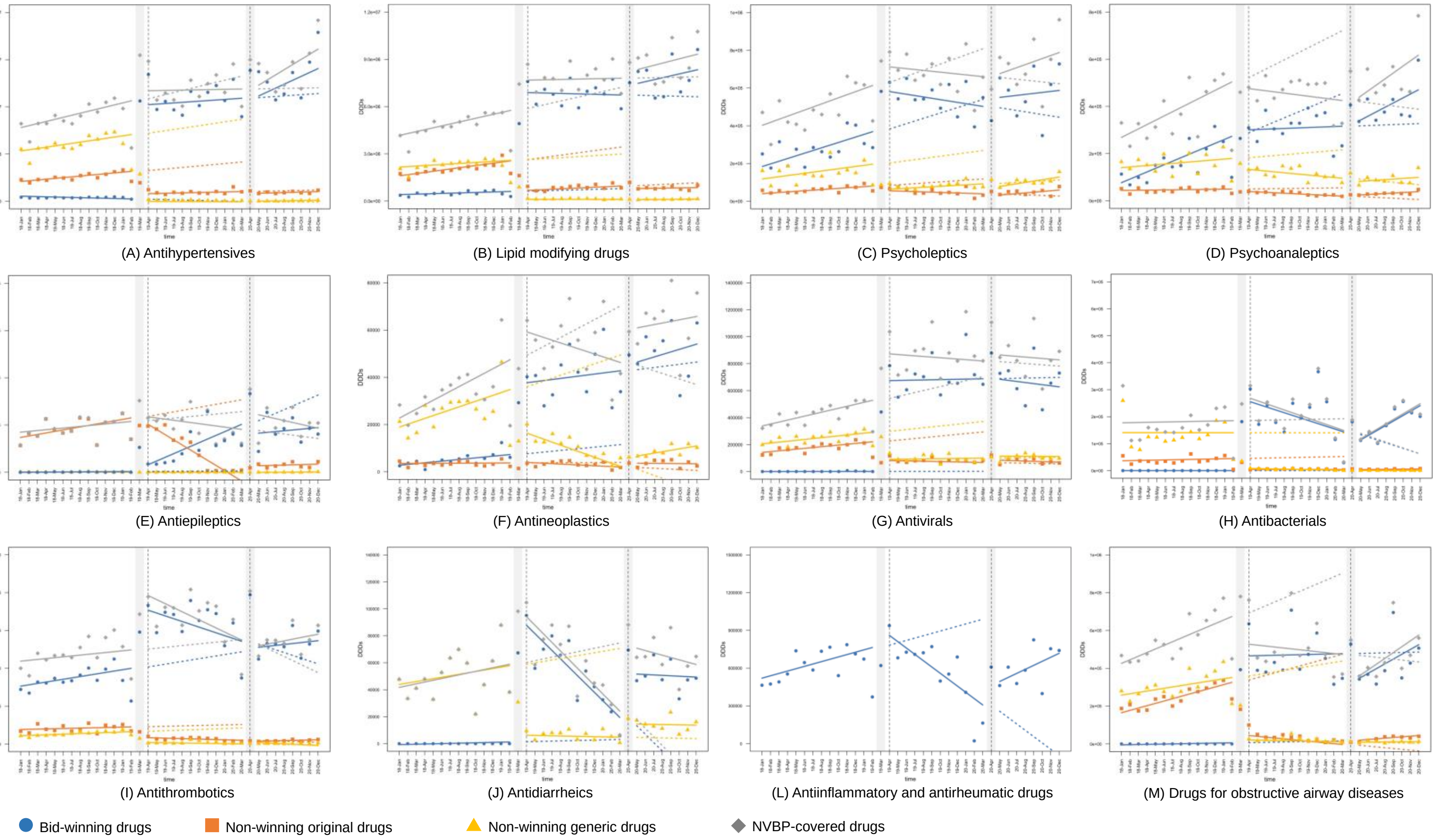

Figure S4. Subgroup Analyses: Procurement Volume of Bid-winning, Non-winning, and NVBP covered Drugs before and after the Pilot NVBP

Note: 1. Shaded area represents that data in this month was excluded in this study. Vertical dashed line represents that the intervention was implemented in this month. 2. Solid line is fitted based on actual values. Dotted line simulates the counterfactual scenario where the intervention was not implemented. 3. NVBP is short for “National Volume-based Procurement”.

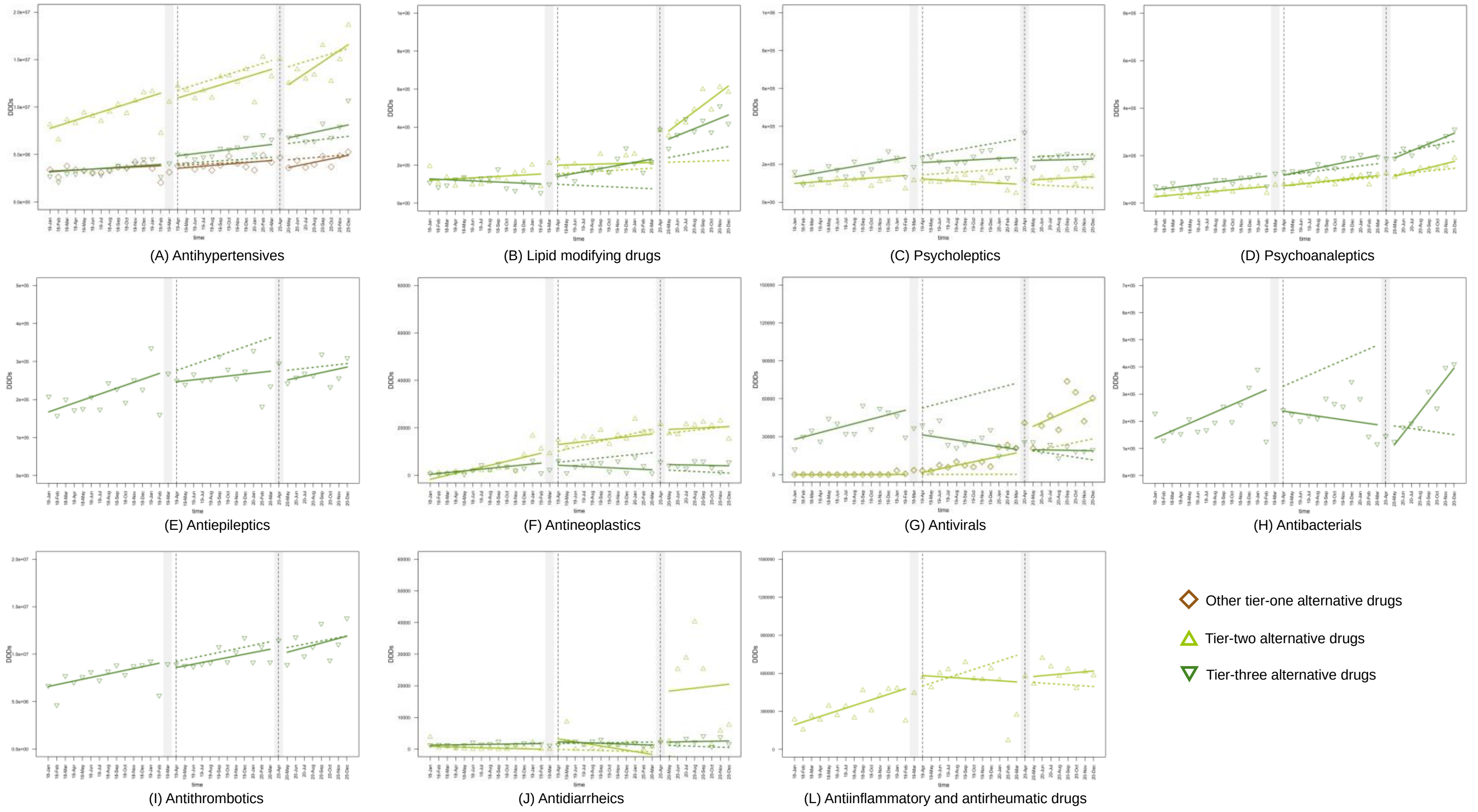

Figure S5. Subgroup Analyses: Procurement Volume of Alternative Drugs before and after the Pilot NVBP

Note: 1. Shaded area represents that data in this month was excluded in this study. Vertical dashed line represents that the intervention was implemented in this month. 2. Solid line is fitted based on actual values. Dotted line simulates the counterfactual scenario where the intervention was not implemented. 3. NVBP is short for "National Volume-based Procurement".

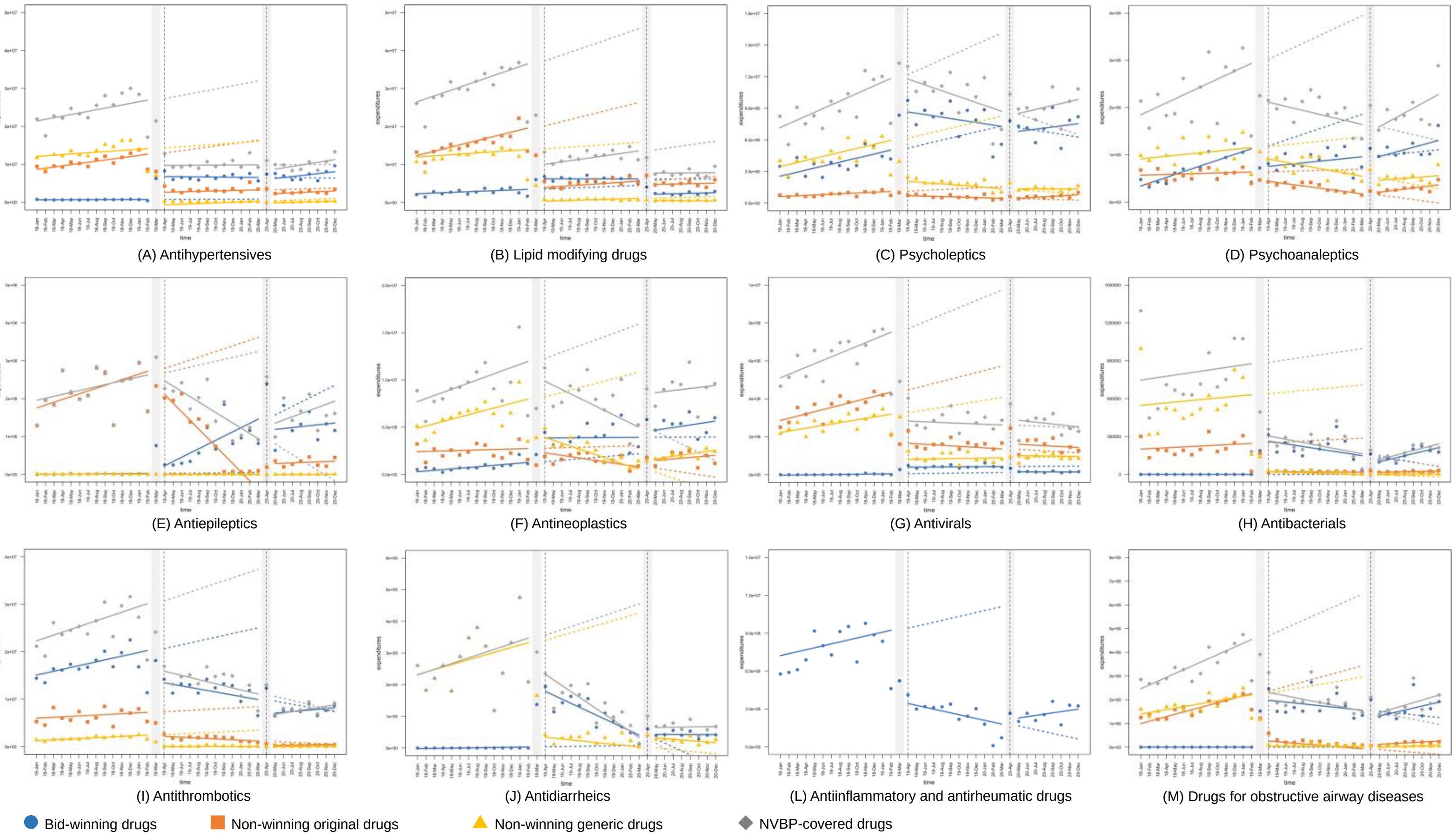

Figure S6. Subgroup Analyses: Procurement Expenditure of Bid-winning, Non-winning, and NVBP covered Drugs before and after the Pilot NVBP

Note: 1. Shaded area represents that data in this month was excluded in this study. Vertical dashed line represents that the intervention was implemented in this month. 2. Solid line is fitted based on actual values. Dotted line simulates the counterfactual scenario where the intervention was not implemented. 3. NVBP is short for “National Volume-based Procurement”.

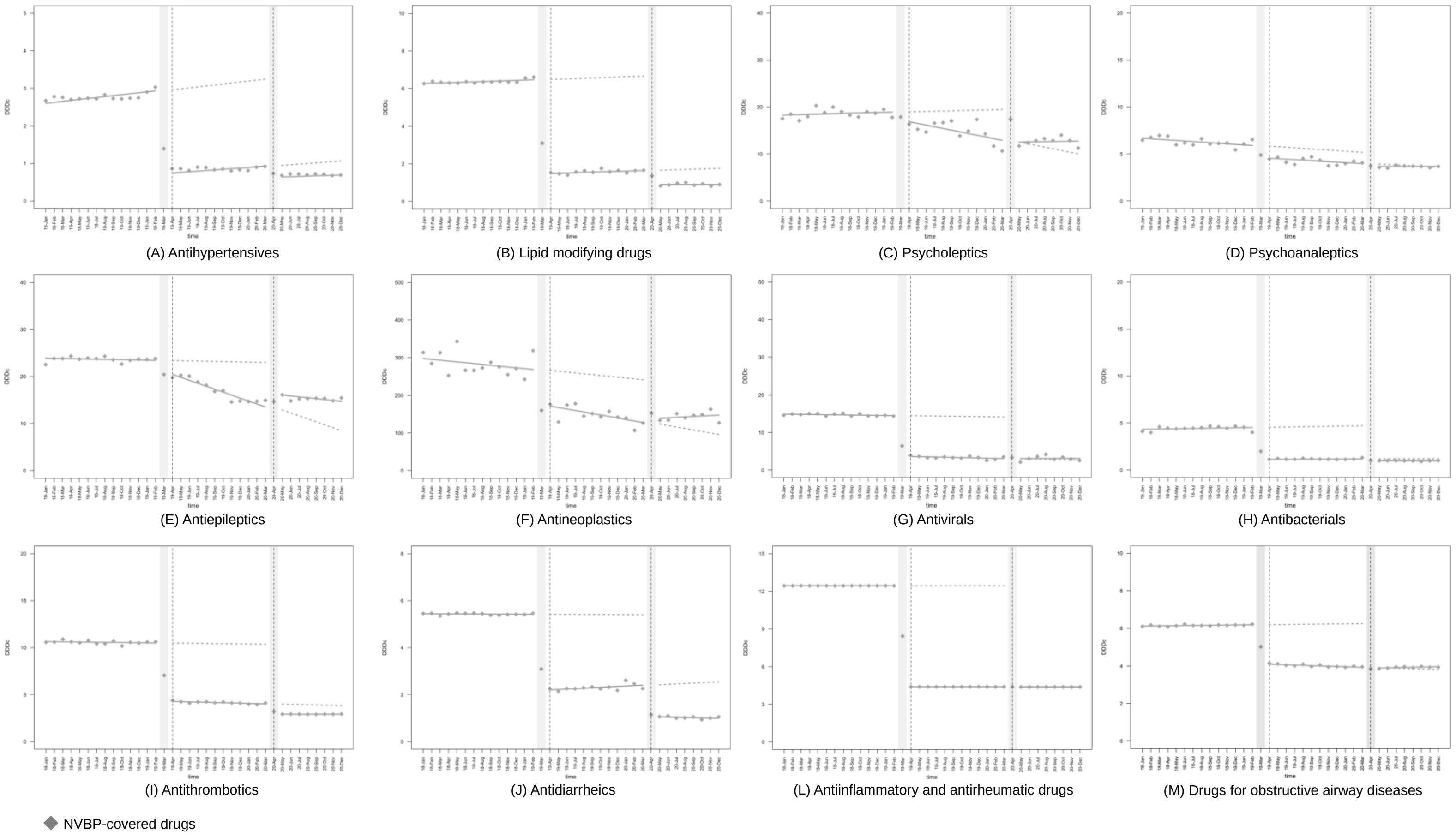

Figure S7. Subgroup Analyses: Procurement Expenditure of Alternative Drugs before and after the Pilot NVBP

Note: 1. Shaded area represents that data in this month was excluded in this study. Vertical dashed line represents that the intervention was implemented in this month. 2. Solid line is fitted based on actual values. Dotted line simulates the counterfactual scenario where the intervention was not implemented. 3. NVBP is short for “National Volume-based Procurement”.
