# Supplementary material for: Impact of China’s National Volume-Based Procurement on Drug Procurement Price, Volume, and Expenditure: An Interrupted Time Series Analysis in Tianjin
Source: Int J Health Policy Manag. 2023 Sep 20;12:7724. doi: 10.34172/ijhpm.2023.7724 (PMC10590231; doi:10.34172/ijhpm.2023.7724)
Supplement: Supplementary file 3 — Supplementary Table of the Subgroup Analysis Results for Procurement Price (Table S5). [file ijhpm-12-7724-s003.pdf]

Article title: Impact of China's National Volume-Based Procurement on Drug Procurement Price, Volume, and Expenditure: An Interrupted Time Series Analysis in Tianjin

Journal name: International Journal of Health Policy and Management (IJHPM)

Authors' information: Boya Zhao[1,2], Jing Wu[1,2]*

[1]School of Pharmaceutical Science and Technology, Tianjin University, Tianjin, China.

[2]Center for Social Science Survey and Data, Tianjin University, Tianjin, China.

***Correspondence to**: Jing Wu, Email: jingwu@tju.edu.cn

Citation: Zhao B, Wu J. Impact of China's national volume-based procurement on drug procurement price, volume, and expenditure: an interrupted time series analysis in Tianjin. Int J Health Policy Manag. 2023;12:7724. doi:10.34172/ijhpm.2023.7724

Supplementary file 3. Supplementary Table of the Subgroup Analysis Results for Procurement Price (Table S5)

Main Content:

Table S5 (Page 2-5). Subgroup Analysis: Effect of the Pilot NVBP on Procurement Price (Results from ITS Analysis)

Note: The testing results of autocorrelation and stationary series are available by contacting the authors.

Table S5. Subgroup Analysis: Effect of the Pilot NVBP on Procurement Price (Results from ITS Analysis)

	Before the pilot NVBP		In the First procurement cycle			In the Second procurement cycle		
	Intercept (β_0)	Trend (β_1)	Level change (β_2)	Trend change (β_3)	Relative change	Level change (β_4)	Trend change (β_5)	Relative change
**Antihypertensives**								
NVBP-covered drugs	2.574 (P<0.001)	0.026 (P<0.001)	-2.210 (P<0.001)	-0.009 (P=0.009)	-72.99%	-0.349 (P<0.001)	-0.009 (P=0.183)	-35.55%
Other tier-one alternative drugs	3.295 (P<0.001)	0.013 (P<0.001)	-0.010 (P=-0.704)	0.003 (P=0.352)	0.00%	-0.048 (P=0.125)	-0.010 (P=0.090)	0.00%
Tier-two alternative drugs	2.666 (P<0.001)	0.006 (P=0.070)	0.023 (P=0.597)	-0.019 (P=0.002)	-3.21%	0.023 (P=0.649)	0.006 (P=0.523)	0.00%
Tier-three alternative drugs	2.296 (P<0.001)	0.019 (P=0.009)	0.148 (P=0.089)	-0.014 (P=0.215)	0.00%	-0.012 (P=0.905)	0.010 (P=0.567)	0.00%
**Lipid-modifying drugs**								
NVBP-covered drugs	6.246 (P<0.001)	0.016 (P=0.005)	-5.009 (P<0.001)	-0.001 (P=0.901)	-76.29%	-0.776 (P<0.001)	-0.015 (P=0.277)	-48.54%
Other tier-one alternative drugs	——	——	——	——	——	——	——	——
Tier-two alternative drugs	4.462 (P<0.001)	0.008 (P=0.234)	0.102 (P=0.223)	-0.037 (P=0.002)	-1.37%	-0.387 (P<0.001)	0.039 (P=0.033)	-8.40%
Tier-three alternative drugs	4.225 (P<0.001)	0.025 (P<0.001)	0.326 (P<0.001)	-0.051 (P<0.001)	0.48%	-0.030 (P=0.742)	0.032 (P=0.059)	4.65%
**Psycholeptics**								
NVBP-covered drugs	18.243 (P<0.001)	0.047 (P=0.586)	-1..664 (P=0.133)	-0.407 (P=0.006)	-19.02%	-0.496 (P=0.688)	0.389 (P=0.096)	0.00%
Other tier-one alternative drugs	——	——	——	——	——	——	——	——

Tier-two alternative drugs	8.571 (P<0.001)	-0.029 (P=0.656)	-0.536 (P=0.518)	0.210 (P=0.054)	0.00%	-0.855 (P=0.367)	-0.253 (P=0.152)	0.00%
Tier-three alternative drugs	10.11 (P<0.001)	-0.160 (P=0.047)	0.377 (P=0.700)	0.235 (P=0.068)	0.00%	0.238 (P=0.831)	-0.014 (P=0.947)	0.00%
**Psychoanaleptics**								
NVBP-covered drugs	6.750 (P<0.001)	-0.060 (P<0.001)	-1.306 (P<0.001)	0.010 (P=0.687)	-24.60%	-0.149 (P=0.542)	-0.045 (P=0.317)	0.00%
Other tier-one alternative drugs	——	——	——	——	——	——	——	——
Tier-two alternative drugs	14.215 (P<0.001)	-0.067 (P<0.001)	-0.369 (P=0.004)	0.034 (P=0.058)	-1.28%	0.101 (P=0.653)	-0.034 (P=0.469)	0.00%
Tier-three alternative drugs	11.218 (P<0.001)	0.019 (P=0.583)	-0.496 (P=0.268)	-0.034 (P=0.552)	0.00%	0.619 (P=0.227)	-0.076 (P=0.421)	0.00%
**Antiepileptics**								
NVBP-covered drugs	23.926 (P<0.001)	-0.037 (P=0.051)	-2.337 (P<0.001)	-0.594 (P<0.001)	-27.04%	2.834 (P<0.001)	0.432 (P<0.001)	51.66%
Other tier-one alternative drugs	——	——	——	——	——	——	——	——
Tier-two alternative drugs	——	——	——	——	——	——	——	——
Tier-three alternative drugs	10.001 (P<0.001)	0.008 (P=0.621)	0.136 (P=0.520)	-0.031 (P=0.211)	0.00%	0.215 (P=0.559)	-0.008 (P=0.892)	0.00%
**Antineoplastics**								
NVBP-covered drugs	299.890 (P<0.001)	-2.251 (P=0.135)	-92.889 (P<0.001)	-1.749 (P=0.463)	-44.12%	14.507 (P=0.495)	5.216 (P=0.188)	0.00%
Other tier-one alternative drugs	——	——	——	——	——	——	——	——

Tier-two alternative drugs	201.607 (P<0.001)	-0.829 (P<0.001)	4.077 (P=0.070)	0.118 (P=0.685)	0.00%	-1.666 (P=0.494)	0.968 (P=0.045)	0.00%
Tier-three alternative drugs	270.588 (P<0.001)	-7.758 (P<0.001)	50.052 (P=0.002)	3.822 (P=0.028)	58.99%	32.069 (P=0.218)	-2.690 (P=0.500)	0.00%
**Antivirals**								
NVBP-covered drugs	14.839 (P<0.001)	-0.030 (P=0.282)	-10.776 (P<0.001)	-0.024 (P=0.595)	-76.33%	0.060 (P=0.880)	0.062 (P=0.400)	0.00%
Other tier-one alternative drugs	39.020 (P<0.001)	0.073 (P=0.847)	5.929 (P=0.191)	-2.056 (P=0.003)	-18.94%	-3,351 (P=0.480)	1.836 (P=0.060)	0.00%
Tier-two alternative drugs	39.020 (P<0.001)	0.073 (P=0.847)	5.929 (P=0.191)	-2.056 (P=0.003)	-18.94%	-3,351 (P=0.480)	1.836 (P=0.060)	0.00%
Tier-three alternative drugs	23.977 (P<0.001)	-0.241 (P<0.001)	1.674 (P=0.001)	0.278 (P<0.001)	17.88%	-0.905 (P=0.155)	0.089 (P=0.498)	0.00%
**Antibacterials**								
NVBP-covered drugs	4.300 (P<0.001)	0.016 (P=0.120)	-3.382 (P<0.001)	-0.013 (P=0.434)	-73.27%	-0.221 (P=0.138)	-0.006 (P=0.830)	0.00%
Other tier-one alternative drugs	——	——	——	——	——	——	——	——
Tier-two alternative drugs	——	——	——	——	——	——	——	——
Tier-three alternative drugs	13.413 (P<0.001)	0.081 (P=0.003)	0.617 (P=0.090)	-0.124 (P=0.001)	-6.10%	-0.069 (P=0.893)	0.036 (P=0.629)	0.00%
**Antithrombotics**								
NVBP-covered drugs	10.654 (P<0.001)	-0.012 (P=0.178)	-6.187 (P<0.001)	-0.013 (P=0.356)	-59.95%	-1.088 (P<0.001)	0.025 (P=0.266)	-26.78%
Other tier-one alternative drugs	——	——	——	——	——	——	——	——

Tier-two alternative drugs	——	——	——	——	——	——	——	——
Tier-three alternative drugs	0.703 (P<0.001)	-0.002 (P=0.148)	0.024 (P=0.228)	0.002 (P=0.480)	0.00%	0.039 (P=0.095)	0.000 (P=0.968)	0.00%
**Antidiarrheics**								
NVBP-covered drugs	5.440 (P<0.001)	-0.002 (P=0.762)	-3.233 (P<0.001)	0.019 (P=0.024)	-57.23%	-1.336 (P<0.001)	-0.026 (P=0.061)	-59.37%
Other tier-one alternative drugs	——	——	——	——	——	——	——	——
Tier-two alternative drugs	0.805 (P=0.006)	0.023 (P=0.471)	0.017 (P=0.966)	-0.046 (P=0.367)	-16.29%	0.211 (P=0.643)	0.380 (P<0.001)	251.23%
Tier-three alternative drugs	3.547 (P<0.001)	0.000 (P=0.233)	-0.001 (P=0.892)	0.001 (P=0.314)	0.00%	-0.002 (P=0.665)	0.000 (P=0.872)	0.00%
**Anti-inflammatory/antirheumatic drugs**								
NVBP-covered drugs	12.439 (P<0.001)	0.000 (P=0.857)	-8.050 (P<0.001)	0.000 (P=0.911)	-64.71%	-0.010 (P<0.001)	0.000 (P=1.000)	-0.23%
Other tier-one alternative drugs	——	——	——	——	——	——	——	——
Tier-two alternative drugs	5.008 (P<0.001)	-0.071 (P=0.024)	0.443 (P=0.244)	0.014 (P=0.765)	0.00%	1.359 (P=0.003)	0.077 (P=0.336)	42.85%
Tier-three alternative drugs	——	——	——	——	——	——	——	——
**Drugs for obstructive airway diseases**								
NVBP-covered drugs	6.115 (P<0.001)	0.005 (P=0.052)	-2.078 (P<0.001)	-0.021 (P<0.001)	-17.15%	-0.056 (P=0.150)	0.026 (P=0.001)	11.55%
Other tier-one alternative drugs	——	——	——	——	——	——	——	——

Tier-two alternative drugs	——	——	——	——	——	——	——	——
Tier-three alternative drugs	——	——	——	——	——	——	——	——

Note:

1. NVBP-covered drugs consist of bid-winning and non-winning drugs.

2. Relative change in this table refers to the relative change at sixth month after the intervention.

3. NVBP is short for "National Volume-based Procurement".
