# Supplementary material for: Impact of China’s National Volume-Based Procurement on Drug Procurement Price, Volume, and Expenditure: An Interrupted Time Series Analysis in Tianjin
Source: Int J Health Policy Manag. 2023 Sep 20;12:7724. doi: 10.34172/ijhpm.2023.7724 (PMC10590231; doi:10.34172/ijhpm.2023.7724)
Supplement: Supplementary file 4 — Supplementary Table of the Subgroup Analysis Results for Procurement Volume (Table S6). [file ijhpm-12-7724-s004.pdf]

Article title: Impact of China's National Volume-Based Procurement on Drug Procurement Price, Volume, and Expenditure: An Interrupted Time Series Analysis in Tianjin

Journal name: International Journal of Health Policy and Management (IJHPM)

Authors' information: Boya Zhao[1,2], Jing Wu[1,2]*

[1]School of Pharmaceutical Science and Technology, Tianjin University, Tianjin, China.

[2]Center for Social Science Survey and Data, Tianjin University, Tianjin, China.

***Correspondence to**: Jing Wu, Email: jingwu@tju.edu.cn

Citation: Zhao B, Wu J. Impact of China's national volume-based procurement on drug procurement price, volume, and expenditure: an interrupted time series analysis in Tianjin. Int J Health Policy Manag. 2023;12:7724. doi:10.34172/ijhpm.2023.7724

Supplementary file 4. Supplementary Table of the Subgroup Analysis Results for Procurement Volume (Table S6)

Main Content:

Table S6 (Page 2-6). Subgroup Analysis: Effect of the Pilot NVBP on Procurement Volume (Results from ITS Analysis)

Note: The testing results of autocorrelation and stationary series are available by contacting the authors.

Table S6. Subgroup Analysis: Effect of the Pilot NVBP on Procurement Volume (Results from ITS Analysis)

	Before the pilot NVBP		In the First procurement cycle			In the Second procurement cycle		
	Intercept (β_0)	Trend (β_1)	Level change (β_2)	Intercept (β_0)	Trend (β_1)	Level change (β_2)	Intercept (β_0)	Trend (β_1)
**Antihypertensives**								
Bid-winning drugs	564,408 (P=0.001)	-22,655 (P=0.227)	9,924,489 (P<0.001)	84,637 (P=0.046)	2,686.63 %	-126,642 (P=0.787)	352,289 (P<0.001)	19.96%
Non-winning original drugs	2,014,420 (P<0.001)	82,557 (P<0.001)	-2,382,911 (P<0.001)	-62,094 (P=0.011)	-76.65%	-372,184 (P=0.057)	4,202 (P=0.902)	0.00%
Non-winning generic drugs	5,199,779 (P<0.001)	133,818 (P<0.001)	-7,084,537 (P<0.001)	-137,139 (P<0.001)	-99.96%	-216,507 (P=0.342)	16,388 (P=0.670)	0.00%
NVBP-covered drugs	7,595,675 (P<0.001)	217,852 (P=0.100)	1,050,661 (P=0.477)	-201,564 (P=0.297)	0.00%	-572,256 (P=0.717)	528,777 (P=0.073)	0.00%
Other tier-one alternative drugs	3,207,470 (P<0.001)	43,604 (P=0.206)	-345,059 (P=0.377)	32,005 (P=0.526)	0.00%	-1,014,408 (P=0.021)	107,129 (P=0.162)	0.00%
Tier-two alternative drugs	7,470,037 (P<0.001)	285,608 (P=0.003)	-786,731 (P=0.425)	-11,470 (P=0.928)	0.00%	-2,776,912 (P=0.013)	326,897 (P=0.094)	-3.74%
Tier-three alternative drugs	3,109,930 (P<0.001)	61,413 (P<0.001)	798,417 (P<0.001)	46,219 (P<0.001)	24.80%	390,028 (P=0.002)	85,935 (P<0.001)	13.51%
**Lipid-modifying drugs**								
Bid-winning drugs	395,690 (P=0.441)	15,369 (P=804)	6,289,993 (P<0.001)	-29,266 (P=0.750)	1,346.83 %	586,151 (P=0.442)	137,296 (P=0.321)	0.00%
Non-winning original drugs	1,535,897 (P<0.001)	72,915 (P<0.001)	-1,903,008 (P<0.001)	-47,538 (P=0.012)	-74.28%	-316,013 (P=0.039)	-16,812 (P=0.527)	-33.01%
Non-winning generic drugs	2,127,427 (P<0.001)	33,027 (P=0.001)	-2,478,278 (P<0.001)	-33,399 (P=0.013)	-95.88%	-89,526 (P=0.392)	6,296 (P=0.737)	0.00%
NVBP-covered drugs	4,059,014 (P<0.001)	121,312 (P=0.082)	1,908,707 (P=0.020)	-110,202 (P=0.278)	31.31%	180,613 (P=0.827)	126,780 (P=0.400)	0.00%
Other tier-one alternative drugs	——	——	——	——	——	——	——	——
Tier-two alternative drugs	118,184 (P<0.001)	2,525 (P=0.421)	44,633 (P=0.216)	-1,186 (P=0.797)	0.00%	126,807 (P=0.003)	32,415 (P<0.001)	82.49%
Tier-three alternative drugs	130,135 (P<0.001)	2,061 (P=0.537)	31,118 (P=0.416)	10,336 (P=0.044)	72.34%	91,773 (P=0.032)	9,786 (P=0.191)	48.14%
**Psycholeptics**								
Bid-winning drugs	170,140 (P<0.001)	14,184 (P=0.012)	219,554 (P<0.001)	-21,337 (P=0.013)	34.56%	14,281 (P=0.817)	12,417 (P=0.283)	0.00%

Non-winning original drugs	37,294 (P<0.001)	3,114 (P<0.001)	-23,909 (P<0.001)	-4,576 (P<0.001)	-51.59%	-16,385 (P<0.001)	5,237 (P<0.001)	49.04%
Non-winning generic drugs	112,339 (P<0.001)	6,026 (P<0.001)	-135,248 (P<0.001)	-3,455 (P=0.067)	-66.98%	-32,906 (P=0.032)	4,537 (P=0.126)	-5.28%
NVBP-covered drugs	386,409 (P<0.001)	16,220 (P=0.052)	144,504 (P=0.269)	32,727 (P=0.094)	0.00%	67,877 (P=0.744)	32,472 (P=0.255)	0.00%
Other tier-one alternative drugs	——	——	——	——	——	——	——	——
Tier-two alternative drugs	95,941 (P<0.001)	3,217 (P=0.202)	-10,191 (P=0.719)	-7,184 (P=0.060)	0.00%	25,853 (P=0.398)	6,398 (P=0.250)	0.00%
Tier-three alternative drugs	125,499 (P<0.001)	7,902 (P=0.004)	-29,279 (P=0.316)	- 5,512 (P=0.156)	0.00%	-32,086 (P=0.309)	-1,324 (P=0.814)	0.00%
**Psychoanaleptics**								
Bid-winning drugs	61,763 (P=0.130)	15,091 (P=0.004)	26,364 (P=0.636)	-13,709 (P=0.070)	0.00%	-26,465 (P=0.660)	17,404 (P=0.116)	0.00%
Non-winning original drugs	43,547 (P<0.001)	440 (P=0.410)	-8,312 (P=0.180)	-2,231 (P=0.009)	-29.81%	1,583 (P=0.810)	3,845 (P=0.003)	131.64%
Non-winning generic drugs	137,339 (P<0.001)	3,016 (P=0.071)	-43,054 (P=0.018)	-6,427 (P=0.013)	-25.04%	-21,883 (P=0.244)	5,863 (P=0.099)	0.00%
NVBP-covered drugs	250,501 (P<0.001)	18,069 (P=0.009)	-23,287 (P=0.754)	-22,662 (P=0.027)	-28.03%	-48,519 (P=0.546)	30,121 (P=0.045)	62.72%
Other tier-one alternative drugs	——	——	——	——	——	——	——	——
Tier-two alternative drugs	24,771 (P=0.002)	3,240 (P=0.001)	-641 (P=0.950)	643 (P=0.633)	0.00%	-17,629 (P=0.119)	5,015 (P=0.017)	16.49%
Tier-three alternative drugs	52,860 (P<0.001)	4,360 (P=0.001)	-3,178 (P=0.821)	3,156 (P=0.097)	0.00%	-33,411 (P=0.035)	7,297 (P=0.012)	18.19%
**Antiepileptics**								
Bid-winning drugs	-36 (P=0.996)	108 (P=0.887)	6,590 (P=0.422)	7,663 (P<0.001)	29 682.15%	-20,488 (P=0.039)	-6,087 (P=0.007)	-34.05%
Non-winning original drugs	70,407 (P<0.001)	3,187 (P=0.018)	-2,392 (P=0.869)	-14,091 (P<0.001)	-60.03%	25,091 (P=0.119)	11,394 (P<0.001)	-99.99%
Non-winning generic drugs	788 (P=0.008)	-37 (P=0.274)	-230 (P=0.552)	37 (P=0.465)	0.00%	-385 (P=0.359)	157 (P=0.044)	0.00%
NVBP-covered drugs	83,077 (P<0.001)	1,743 (P=0.004)	12,345 (P=0.013)	-4,161 (P<0.001)	-11/73%	31,452 (P<0.001)	-1,640 (P=0.405)	38.24%
Other tier-one alternative drugs	——	——	——	——	——	——	——	——
Tier-two alternative drugs	——	——	——	——	——	——	——	——

Tier-three alternative drugs	159,681 (P<0.001)	7,799 (P=0.007)	-24,648 (P=0.421)	-5,283 (P=0.195)	0.00%	-43,237 (P=0.196)	2,316 (P=0.696)	0.00%
**Antineoplastics**								
Bid-winning drugs	2,441 (P=0.132)	348 (P=0.097)	29,936 (P<0.001)	111 (P=0.737)	267.45%	1,924 (P=0.619)	626 (P=0.339)	13.58%
Non-winning original drugs	3,501 (P<0.001)	18 (P=0.848)	405 (P=0.708)	-197 (P=0.169)	0.00%	1,595 (P=0.176)	148 (P=0.482)	0.00%
Non-winning generic drugs	17,940 (P<0.001)	1,170 (P<0.001)	-15,449 (P<0.001)	-2,634 (P<0.001)	-75.35%	3,373 (P=0.230)	1,960 (P=0.001)	+
NVBP-covered drugs	20,883 (P<0.001)	1,898 (P<0.001)	12,902 (P<0.001)	-3,073 (P<0.001)	-4.79%	10,220 (P=0.055)	1,862 (P=0.070)	26.90%
Other tier-one alternative drugs	——	——	——	——	——	——	——	——
Tier-two alternative drugs	-3,851 (P<0.001)	845 (P<0.001)	3,188 (P<0.001)	-158 (P=0.241)	17.91%	286 (P=0.801)	-839 (P=0.001)	-20.78%
Tier-three alternative drugs	52 (P=0.924)	365 (P<0.001)	-720 (P=0.269)	-539 (P<0.001)	-49.35%	1 537 (P=0.104)	101 (P=0.542)	0.00%
**Antivirals**								
Bid-winning drugs	28 (P=0.999)	51 (P=0.991)	672,304 (P<0.001)	1,202 (P=0.862)	224 758.87%	3,970 (P=0.940)	-9,283 (P=0.362)	0.00%
Non-winning original drugs	135,744 (P<0.001)	6,073 (P<0.001)	-137,442 (P<0.001)	-7,436 (P<0.001)	-71.01%	7,276 (P=0.540)	-782 (P=0.713)	0.00%
Non-winning generic drugs	198,328 (P<0.001)	6,648 (P<0.001)	-201,802 (P<0.001)	-5,808 (P<0.001)	-73.48%	1,526 (P=0.843)	-1,212 (P=0.414)	0.00%
NVBP-covered drugs	323,687 (P<0.001)	14,600 (P=0.136)	349,037 (P=0.004)	-19,368 (P=0.180)	40.70%	20,572 (P=0.861)	-448 (P=0.983)	0.00%
Other tier-one alternative drugs	-42 (P=0.959)	10 (P=0.917)	227 (P=0.749)	1,386 (P<0.001)	45,619.09%	18,020 (P<0.001)	1,620 (P<0.001)	101.52%
Tier-two alternative drugs	-42 (P=0.959)	10 (P=0.917)	227 (P=0.749)	1,386 (P<0.001)	45,619.09%	18,020 (P<0.001)	1,620 (P<0.001)	101.52%
Tier-three alternative drugs	26,156 (P<0.001)	1,768 (P<0.001)	-18,389 (P<0.001)	-2,814 (P<0.001)	-57.48%	-3,715 (P=0.185)	931 (P=0.090)	0.00%
**Antibacterials**								
Bid-winning drugs	0 (P=1.000)	0 (P=1.000)	265,568 (P<0.001)	-10 176 (P=0.143)	+	-47,618 (P=0.400)	28,312 (P=0.009)	164.39%
Non-winning original drugs	35,875 (P<0.001)	632 (P=0.295)	-39,882 (P<0.001)	-693 (P=0.436)	-87.60%	-2,046 (P=0.779)	407 (P=0.758)	0.00%
Non-winning generic drugs	140,874 (P<0.001)	-31 (P=0.990)	-131,822 (P<0.001)	-576 (P=0.880)	-97.72%	-1,218 (P=0.969)	716 (P=0.899)	0.00%
NVBP-covered drugs	176,748 (P<0.001)	601 (P=0.914)	93 864 (P=0.147)	-11,445 (P=0.172)	0.00%	-50,882 (P=0.456)	29,435 (P=0.023)	149.96%

Other tier-one alternative drugs	——	——	——	——	——	——	——	——
Tier-two alternative drugs	——	——	——	——	——	——	——	——
Tier-three alternative drugs	123,651 (P=0.003)	13,685 (P=0.005)	-73,118 (P=0.165)	-18,256 (P=0.011)	-31.56%	-142,324 (P=0.016)	45,007 (P<0.001)	105.65%
**Antithrombotics**								
Bid-winning drugs	1492,691 (P<0.001)	36248 (P=0.141)	1,610,215 (P<0.001)	-110,871 (P=0.001)	27.7%	-258,777 (P=0.364)	99,841 (P=0.062)	0.0%
Non-winning original drugs	356,752 (P<0.001)	8,846 (P=0.002)	-293,135 (P<0.001)	-17,542 (P<0.001)	-66.8%	-49,737 (P=0.158)	17,198 (P=0.015)	0.0%
Non-winning generic drugs	184,569 (P<0.001)	13,450 (P<0.001)	-347,205 (P<0.001)	-12,808 (P<0.001)	-93.9%	-69,699 (P=0.006)	4,063 (P=0.308)	-99.9%
NVBP-covered drugs	2,022,011 (P<0.001)	59,384 (P=0.029)	971,791 (P=0.003)	-142,439 (P<0.001)	-7.1%	-396,549 (P=0.216)	124,646 (P=0.036)	0.0%
Other tier-one alternative drugs	——	——	——	——	——	——	——	——
Tier-two alternative drugs	——	——	——	——	——	——	——	——
Tier-three alternative drugs	6,678,888 (P<0.001)	178,670 (P<0.001)	-424,845 (P=0.066)	-82,949 (P=0.009)	-4.18%	-1,409,002 (P<0.001)	354,677 (P<0.001)	9.32%
**Antidiarrheics**								
Bid-winning drugs	-889 (P=0.811)	154 (P=0.731)	92,751 (P<0.001)	-6,366 (P<0.001)	11.43%	32,134 (P<0.001)	5,890 (P<0.001)	0.13%
Non-winning original drugs	——	——	——	——	——	——	——	——
Non-winning generic drugs	41,035 (P<0.001)	1,457 (P<0.001)	-53,512 (P<0.001)	-1,963 (P<0.001)	-93.30%	13,471 (P=0.001)	-340 (P=0.601)	+
NVBP-covered drugs	39,088 (P<0.001)	1,854 (P=0.004)	40,307 (P<0.001)	-9,087 (P<0.001)	-22.69%	47,043 (P<0.001)	6,221 (P<0.001)	+
Other tier-one alternative drugs	——	——	——	——	——	——	——	——
Tier-two alternative drugs	1,209 (P=0.666)	-84 (P=0.811)	3,632 (P=0.372)	-306 (P=0.549)	0.00%	23,030 (P<0.001)	-225 (P=0.795)	1 ,798.43 %
Tier-three alternative drugs	1,169 (P=0.035)	46 (P=0.478)	645 (P=0.382)	-155 (P=0.111)	0.00%	968 (P=0.224)	157 (P=0.273)	0.00%
**Anti-inflammatory/antirheumatic drugs**								
Bid-winning drugs	501,796 (P<0.001)	18,722 (P=0.088)	194,585 (P=0.119)	-81,606 (P<0.001)	-44.37%	220,904 (P=0.099)	94,850 (P<0.001)	4 ,640.64 %
Non-winning original drugs	——	——	——	——	——	——	——	——

Non-winning generic drugs	——	——	——	——	——	——	——	——
NVBP-covered drugs	501,796 (P<0.001)	18,722 (P=0.088)	194,585 (P=0.119)	-81,606 (P<0.001)	-44.37%	220,904 (P=0.099)	94,850 (P<0.001)	4 640.64%
Other tier-one alternative drugs	——	——	——	——	——	——	——	——
Tier-two alternative drugs	172,266 (P<0.001)	21,867 (P<0.001)	108,423 (P=0.003)	-26,422 (P<0.001)	11.28%	-7,144 (P=0.886)	10,759 (P=0.332)	54.51%
Tier-three alternative drugs	——	——	——	——	——	——	——	——
**Drugs for obstructive airway diseases**								
Bid-winning drugs	0 (P=1.000)	0 (P=1.000)	520,454 (P<0.001)	-9,897 (P=0.403)	+	-76,614 (P=0.432)	31,911 (P=0.078)	0.00%
Non-winning original drugs	133,473 (P<0.001)	14,664 (P<0.001)	-287,494 (P<0.001)	-19,894 (P<0.001)	-95.36%	-1,195 (P=0.762)	8,732 (P<0.001)	+
Non-winning generic drugs	232,379 (P<0.001)	11,720 (P<0.001)	-379,442 (P<0.001)	-12,452 (P=0.001)	-96.80%	-22,027 (P=0.431)	1,035 (P=0.837)	0.00%
NVBP-covered drugs	371,695 (P<0.001)	25,345 (P<0.001)	-184,768 (P=0.001)	-31,040 (P=0.000)	-43.07%	-207,905 (P=0.006)	39,330 (P=0.003)	12.67%
Other tier-one alternative drugs	——	——	——	——	——	——	——	——
Tier-two alternative drugs	——	——	——	——	——	——	——	——
Tier-three alternative drugs	——	——	——	——	——	——	——	——

Note:

1. NVBP-covered drugs consist of Bid-winning and Non-winning drugs.

2. Relative change in this table refers to the relative change at the sixth month after the intervention.

3. NVBP is short for “National Volume-based Procurement”.
