# Supplementary material for: Impact of China’s National Volume-Based Procurement on Drug Procurement Price, Volume, and Expenditure: An Interrupted Time Series Analysis in Tianjin
Source: Int J Health Policy Manag. 2023 Sep 20;12:7724. doi: 10.34172/ijhpm.2023.7724 (PMC10590231; doi:10.34172/ijhpm.2023.7724)
Supplement: Supplementary file 5 — Supplementary Table of the Subgroup Analysis Results for Procurement Expenditure (Table S7). [file ijhpm-12-7724-s005.pdf]

Article title: Impact of China's National Volume-Based Procurement on Drug Procurement Price, Volume, and Expenditure: An Interrupted Time Series Analysis in Tianjin

Journal name: International Journal of Health Policy and Management (IJHPM)

Authors' information: Boya Zhao[1,2], Jing Wu[1,2]*

[1]School of Pharmaceutical Science and Technology, Tianjin University, Tianjin, China.

[2]Center for Social Science Survey and Data, Tianjin University, Tianjin, China.

***Correspondence to**: Jing Wu, Email: jingwu@tju.edu.cn

Citation: Zhao B, Wu J. Impact of China's national volume-based procurement on drug procurement price, volume, and expenditure: an interrupted time series analysis in Tianjin. Int J Health Policy Manag. 2023;12:7724. doi:10.34172/ijhpm.2023.7724

Supplementary file 5. Supplementary Table of the Subgroup Analysis Results for Procurement Expenditure (Table S7)

Main Content:

Table S7 (Page 2-8). Subgroup Analysis: Effect of the Pilot NVBP on Procurement Expenditure (Results from ITS Analysis)

Note: The testing results of autocorrelation and stationary series are available by contacting the authors.

Table S6. Subgroup Analysis: Effect of the Pilot NVBP on Procurement Expenditure (Results from ITS Analysis)

	Before the pilot NVBP		In the First procurement cycle			In the Second procurement cycle		
	Intercept (β_0)	Trend (β_1)	Level change (β_2)	Intercept (β_0)	Trend (β_1)	Level change (β_2)	Intercept (β_0)	Trend (β_1)
**Antihypertensives**								
Bid-winning drugs	638,990 (P=0.244)	6,116 (P=0.926)	6,074,294 (P<0.001)	-26,612 (P=0.785)	897.44%	-526,422 (P=0.514)	269,576 (P=0.072)	13.99%
Non-winning original drugs	7,048,883 (P<0.001)	492,551 (P<0.001)	-12,289 777 (P<0.001)	-281,799 (P<0.001)	-82.78%	-3,394,498 (P<0.001)	18,113 (P=0.669)	-61.18%
Non-winning generic drugs	11,217,411 (P<0.001)	337,837 (P<0.001)	-16 228 792 (P<0.001)	-324,966 (P<0.001)	-99.99%	-586,917 (P=0.136)	21,290 (P=0.749)	0.00%
NVBP-covered drugs	20,243,612 (P<0.001)	684,308 (P<0.001)	- 20,470,262 (P<0.001)	-642,083 (P=0.001)	-72.24%	-2,863,892 (P=0.059)	316,845 (P=0.238)	0.00%
Other tier-one alternative drugs	10,544,743 (P<0.001)	191,924 (P=0.121)	-1,233,107 (P=0.375)	141,143 (P=0.433)	0.00%	-3,832,346 (P=0.015)	365,899 (P=0.178)	0.00%
Tier-two alternative drugs	19,548 270 (P<0.001)	830,673 (P<0.001)	-2,109,800 (P=0.089)	-159,985 (P=0.378)	-8.70%	-5,735,511 (P<0.001)	429,304 (P=0.112)	-13.62%
Tier-three alternative drugs	5,494,073 (P<0.001)	403,088 (P<0.001)	1,117,894 (P=0.148)	20,855 (P=0.774)	0.00%	-470,776 (P=0.510)	472,390 (P=0.006)	11.90%
**Lipid-modifying drugs**								
Bid-winning drugs	2,244,584 (P<0.001)	86,654 (P=0.064)	2,772,293 (P<0.001)	-88,062 (P=0.196)	118.41%	-4 236 759 (P<0.001)	55 873 (P=0.577)	-62.02%
Non-winning original drugs	11,786 557 (P<0.001)	557,540 (P<0.001)	- 15,805,704 (P<0.001)	-402,433 (P=0.003)	-79.69%	-2 031 734 (P=0.051)	-108 544 (P=0.550)	-34.98%
Non-winning generic drugs	11,816,556 (P<0.001)	153,481 (P<0.001)	- 13,668,109 (P<0.001)	-87,826 (P<0.001)	-96.38%	-805,456 (P<0.001)	-85,833 (P<0.001)	-83.76%
NVBP-covered drugs	25,702,778 (P<0.001)	768,599 (P<0.001)	- 26,731,145 (P<0.001)	-449,385 (P<0.001)	-72.05%	-7,372,475 (P<0.001)	-303,951 (P=0.001)	-57.42%
Other tier-one alternative drugs	——	——	——	——	——	——	——	——
Tier-two alternative drugs	527,184 (P<0.001)	12,359 (P=0.329)	224,396 (P=0.126)	-11,346 (P=0.543)	0.00%	396,326 (P=0.015)	140,329 (P<0.001)	93.83%

Tier-three alternative drugs	575,014 (P<0.001)	-5,441 (P=0.001)	124,622 (P<0.001)	50,471 (P<0.001)	63.16%	364,618 (P<0.001)	43,667 (P<0.001)	38.96%
**Psycholeptics**								
Bid-winning drugs	2,356,795 (P<0.001)	190,682 (P=0.011)	3,757,652 (P<0.001)	-316,041 (P=0.006)	116.68%	-955,798 (P=0.256)	229,452 (P=0.142)	0.00%
Non-winning original drugs	578,242 (P<0.001)	37,629 (P=0.004)	-372,954 (P=0.012)	-60,172 (P=0.003)	-52.58%	-125,813 (P=0.404)	69,186 (P=0.015)	176.37%
Non-winning generic drugs	3,271,728 (P<0.001)	191,330 (P<0.001)	-3,803,774 (P<0.001)	-256,595 (P<0.001)	-77.39%	-392,257 (P=0.148)	65,343 (P=0.195)	0.00%
NVBP-covered drugs	6,795,879 (P<0.001)	359,250 (P=0.003)	234,299 (P=0.856)	-637,423 (P=0.001)	-24.32%	-1,118,810 (P=0.425)	467,453 (P=0.071)	0.00%
Other tier-one alternative drugs	——	——	——	——	——	——	——	——
Tier-two alternative drugs	837,386 (P<0.001)	21,622 (P=0.235)	-98,814 (P=0.633)	-41,358 (P=0.136)	0.00%	175,313 (P=0.434)	31,670 (P=0.430)	0.00%
Tier-three alternative drugs	1,224,695 (P<0.001)	49,756 (P=0.053)	-202,237 (P=0.479)	-29,373 (P=0.435)	0.00%	-92,438 (P=0.764)	2,747 (P=0.960)	0.00%
**Psychoanaleptics**								
Bid-winning drugs	279,072 (P<0.001)	60,535 (P<0.001)	-391,118 (P=0.026)	-42,018 (P=0.065)	-44.59%	-168,612 (P=0.354)	30,567 (P=0.350)	0.00%
Non-winning original drugs	563,881 (P<0.001)	5,007 (P=0.465)	-170,442 (P=0.037)	-29,040 (P=0.009)	-45.74%	-2,082 (P=0.980)	46,695 (P=0.005)	116.43%
Non-winning generic drugs	894,774 (P<0.001)	18,525 (P=0.134)	-210,282 (P=0.113)	-51,639 (P=0.009)	-24.92%	-137,327 (P=0.330)	47,117 (P=0.078)	104.39%
NVBP-covered drugs	1,761,564 (P<0.001)	83,718 (P=0.014)	-777,750 (P=0.042)	-126,567 (P=0.014)	-47.82%	-321,989 (P=0.419)	140,555 (P=0.057)	0.00%
Other tier-one alternative drugs	——	——	——	——	——	——	——	——
Tier-two alternative drugs	369,332 (P<0.001)	39,411 (P=0.001)	-30,918 (P=0.794)	7,224 (P=0.642)	0.00%	-189,340 (P=0.146)	45,691 (P=0.055)	0.00%
Tier-three alternative drugs	599,278 (P<0.001)	49,305 (P=0.002)	-68,441 (P=0.672)	30,623 (P=0.158)	0.00%	-232,041 (P=0.191)	61,213 (P=0.060)	0.00%
**Antiepileptics**								
Bid-winning drugs	423 (P=0.996)	1,694 (P=0.876)	92,180 (P=0.434)	110,170 (P<0.001)	17 462.91%	-298,452 (P=0.036)	-87,658 (P=0.007)	-34.21%
Non-winning original drugs	1,683,233 (P<0.001)	74,297 (P=0.021)	-385,142 (P=0.275)	-302,386 (P<0.001)	-51.54%	527,010 (P=0.170)	236,911 (P=0.002)	-129.21%
Non-winning generic drugs	9,474 (P<0.001)	-226 (P=0.152)	-5 943 (P<0.001)	214 (P=0.382)	-92.61%	-1,550 (P=0.465)	611 (P=0.195)	0.00%
NVBP-covered drugs	1,696,145 (P<0.001)	74,657 (P=0.046)	-199,254 (P=0.631)	-216,213 (P=0.001)	-39.92%	486,293 (P=0.282)	161,273 (P=0.053)	0.00%

Other tier-one alternative drugs	——	——	——	——	——	——	——	——
Tier-two alternative drugs	——	——	——	——	——	——	——	——
Tier-three alternative drugs	1,599,622 (P<0.001)	78 762 (P=0.055)	-212 152 (P=0.471)	-54,271 (P=0.167)	0.00%	-341 751 (P=0.286)	-5 053 (P=0.929)	0.00%
**Antineoplastics**								
Bid-winning drugs	205,236 (P=0.379)	76,168 (P=0.015)	2,581,046 (P<0.001)	-70,499 (P=0.142)	173.94%	474,464 (P=0.358)	129,680 (P=0.178)	17.23%
Non-winning original drugs	2,585,034 (P<0.001)	-314 (P=)	-477,807 (P=0.418)	-95,132 (P=0.218)	0.00%	810,018 (P=0.205)	80,335 (P=0.481)	0.00%
Non-winning generic drugs	4,652,535 (P<0.001)	237,129 (P=0.002)	-3,738,480 (P<0.001)	-542,834 (P<0.001)	-75.57%	465,611 (P=0.571)	438,028 (P=0.006)	+
NVBP-covered drugs	7,364,735 (P<0.001)	327,708 (P=0.039)	-1,634,808 (P=0.349)	-768,679 (P=0.002)	-31.15%	2,870,158 (P=0.131)	548,497 (P=0.111)	0.00%
Other tier-one alternative drugs	——	——	——	——	——	——	——	——
Tier-two alternative drugs	-467,141 (P=0.204)	160,867 (P=0.001)	672,647 (P=0.187)	95,322 (P=0.151)	0.00%	-58,779 (P=0.913)	-29,343 (P=0.762)	0.00%
Tier-three alternative drugs	134,104 (P-0.080)	51,956 (P<0.001)	-155,964 (P=0.082)	-77,529 (P<0.001)	-51.98%	191,596 (P=0.130)	8,482 (P=0.700)	0.00%
**Antivirals**								
Bid-winning drugs	-34,201 (P=0.002)	5,657 (P<0.001)	349,076 (P<0.001)	-2,729 (P=0.201)	3,864.28 %	-292,949 (P<0.001)	-3,814 (P=0.224)	-64.34%
Non-winning original drugs	2,744,082 (P<0.001)	114,782 (P=0.001)	-2,673,403 (P<0.001)	-141,127 (P=0.003)	-69.32%	29,569 (P=0.934)	3,033 (P=0.962)	0.00%
Non-winning generic drugs	2,155,065 (P<0.001)	73,783 (P<0.001)	-2,381,247 (P<0.001)	-66,657 (P<0.001)	-76.87%	-28,516 (P=0.706)	-13,241 (P=0.363)	0.00%
NVBP-covered drugs	4,921,650 (P<0.001)	185,321 (P<0.001)	-4,673,532 (P<0.001)	-203,960 (P<0.001)	-67.66%	-71,315 (P=0.789)	-31,230 (P=0.514)	-13.02%
Other tier-one alternative drugs	-40,262 (P=0.001)	5,356 (P<0.001)	99,115 (P<0.001)	22,185 (P<0.001)	364.85%	68,789 (P=0.003)	52,363 (P<0.001)	60.44%
Tier-two alternative drugs	-40,262 (P=0.001)	5,356 (P<0.001)	99,115 (P<0.001)	22,185 (P<0.001)	364.85%	68,789 (P=0.003)	52,363 (P<0.001)	60.44%
Tier-three alternative drugs	588,144 (P<0.001)	36,781 (P=0.001)	-351,582 (P=0.004)	-62,511 (P<0.001)	-53.87%	-40,663 (P=0.735)	15,880 (P=0.466)	0.00%
**Antibacterials**								
Bid-winning drugs	0 (P=1.000)	0 (P=1.000)	273,552 (P<0.001)	-10,467 (P=0.138)	+	-63,789 (P=0.269)	26,421 (P=0.016)	151.14%
Non-winning original drugs	196,892 (P<0.001)	3,470 (P=0.293)	-224,808 (P<0.001)	-3,738 (P=0.442)	-90.04%	-9,700 (P=0.808)	1,792 (P=0.804)	0.00%

Non-winning generic drugs	541,514 (P<0.001)	2,954 (P=0.506)	-491,895 (P<0.001)	-15,061 (P=0.008)	-95.56%	111,272 (P=0.246)	1,524 (P=0.893)	0.00%
NVBP-covered drugs	737,088 (P<0.001)	5,598 (P=0.373)	-322,706 (P<0.001)	-43,600 (P<0.001)	-65.06%	278,231 (P=0.050)	6,964 (P=0.682)	0.00%
Other tier-one alternative drugs	——	——	——	——	——	——	——	——
Tier-two alternative drugs	——	——	——	——	——	——	——	——
Tier-three alternative drugs	1,613,478 (P=0.006)	211,949 (P=0.003)	-843,755 (P=0.270)	-297,335 (P=0.005)	-31.91%	-2,066,307 (P=0.016)	690,604 (P<0.001)	91.68%
**Antithrombotics**								
Bid-winning drugs	14,696,232 (P<0.001)	397,746 (P=0.005)	-6,481,005 (P<0.001)	-718,538 (P=0.001)	-47.7%	-3,802,512 (P=0.024)	476,265 (P=0.107)	-11.8%
Non-winning original drugs	5,886,027 (P<0.001)	97 582 (P=0.198)	-4 968 631 (P<0.001)	-188,495 (P=0.097)	-72.6%	-1,155,238 (P=0.212)	112,366 (P=0.497)	0.0%
Non-winning generic drugs	1,216,215 (P<0.001)	86,456 (P<0.001)	-2,397,953 (P<0.001)	-84,574 (P<0.001)	-99.3%	-207,776 (P=0.089)	4,294 (P=0.841)	0.0%
NVBP-covered drugs	21,702,345 (P<0.001)	602,425 (P<0.001)	-13,778,380 (P<0.001)	-1,041,826 (P<0.001)	-61.5%	-5,758,276 (P=0.001)	723,510 (P=0.015)	-22.6%
Other tier-one alternative drugs	——	——	——	——	——	——	——	——
Tier-two alternative drugs	——	——	——	——	——	——	——	——
Tier-three alternative drugs	4,624,887 (P<0.001)	113,678 (P=0.045)	-323,067 (P=0.604)	-12,005 (P=0.882)	0.00%	-536,067 (P=0.424)	155,403 (P=0.204)	0.00%
**Antidiarrheics**								
Bid-winning drugs	-2,403 (P=0.721)	395 (P=0.627)	188,289 (P<0.001)	-12,936 (P<0.001)	1437,048.40%	1,511 (P=0.890)	12,449 (P<0.001)	+
Non-winning original drugs	——	——	——	——	——	——	——	——
Non-winning generic drugs	224,474 (P<0.001)	7,734 (P<0.001)	-296,171 (P<0.001)	-10,496 (P<0.001)	-94.35%	14,628 (P=0.459)	711 (P=0.836)	0.00%
NVBP-covered drugs	221,696 (P<0.001)	8,964 (P=0.008)	-96,500 (P=0.012)	-27,054 (P<0.001)	-66.70%	12,864 (P=0.781)	18,495 (P=0.021)	+
Other tier-one alternative drugs	——	——	——	——	——	——	——	——
Tier-two alternative drugs	696 (P=0.756)	-40 (P=0.886)	4,639 (P=0.099)	-394 (P=0.382)	0.00%	32,190 (P<0.001)	2,328 (P=0.009)	1,793.71%
Tier-three alternative drugs	4,144 (P=0.035)	162 (P=0.478)	2,283 (P=0.382)	-550 (P=0.111)	0.00%	3,425 (P=0.225)	557 (P=0.274)	0.00%

**Anti-inflammatory/antirheumatic drugs**								
Bid-winning drugs	7,071,130 (P<0.001)	155,259 (P<0.001)	-5,659,040 (P<0.001)	-303,533 (<0.001)	-73.47%	78,794 (P=0.798)	247,368 (P<0.001)	151.55%
Non-winning original drugs	——	——	——	——	——	——	——	——
Non-winning generic drugs	——	——	——	——	——	——	——	——
NVBP-covered drugs	7,071,130 (P<0.001)	155,259 (P<0.001)	-5,659,040 (P<0.001)	-303,533 (P<0.001)	-73.47%	78,794 (P=0.798)	247,368 (P<0.001)	151.55%
Other tier-one alternative drugs	——	——	——	——	——	——	——	——
Tier-two alternative drugs	995,699 (P=0.001)	61,460 (P=0.065)	731,565 (P=0.056)	-115,388 (P=0.024)	9.90%	1,073,094 (P=0.012)	35,534 (P=0.620)	11.26%
Tier-three alternative drugs	——	——	——	——	——	——	——	——
**Drugs for obstructive airway diseases**								
Bid-winning drugs	0 (P=1.000)	0 (P=1.000)	2,017,280 (P<0.001)	-38,359 (P=0.399)	+	-324,262 (P=0.388)	121,838 (P=0.080)	0.00%
Non-winning original drugs	897, 688 (P<0.001)	97,517 (P<0.001)	-1,973,664 (P<0.001)	-127,605 (P<0.001)	-96.24%	-24,578 (P=0.410)	49,732 (P<0.001)	+
Non-winning generic drugs	1,333,024 (P<0.001)	62,497 (P<0.001)	-2,163,229 (P<0.001)	-64,161 (P<0.001)	-98.64%	-163,848 (P=0.145)	13,916 (P=0.452)	0.00%
NVBP-covered drugs	2,306,178 (P<0.001)	159,590 (P<0.001)	-2,161,731 (P<0.001)	-230,496 (P<0.001)	-64.07%	-499,080 (P=0.297)	176,897 (P=0.047)	16.52%
Other tier-one alternative drugs	——	——	——	——	——	——	——	——
Tier-two alternative drugs	——	——	——	——	——	——	——	——
Tier-three alternative drugs	——	——	——	——	——	——	——	——

Note:

1. NVBP-covered drugs consist of bid-winning and non-winning drugs.

2. Relative change in this table refers to the relative change at the sixth month after the intervention. "+" or "-" indicate that the relative change can't be calculated as the values pre- or post- the intervention equal to zero.

3. NVBP is short for "National Volume-based Procurement".
